# Supplementary material for: Inflammatory Resolution Triggers a Prolonged Phase of Immune Suppression through COX-1/mPGES-1-Derived Prostaglandin E2
Source: Cell Rep. 2017 Sep 26;20(13):3162–75. doi: 10.1016/j.celrep.2017.08.098 (PMC5639146; doi:10.1016/j.celrep.2017.08.098)
Supplement: Document S1. Figures S1–S4 [file mmc1.pdf]

**Cell Reports, Volume *20***

# Supplemental Information

# Inflammatory Resolution Triggers a Prolonged Phase of Immune Suppression through COX-1/mPGES-1-Derived Prostaglandin $E_2$

**Justine Newson, Madhur P. Motwani, Alexandra C. Kendall, Anna Nicolaou, Giulio G. Muccioli, Mireille Alhouayek, Melanie Bennett, Rachel Van De Merwe, Sarah James, Roel P.H. De Maeyer, and Derek W. Gilroy**

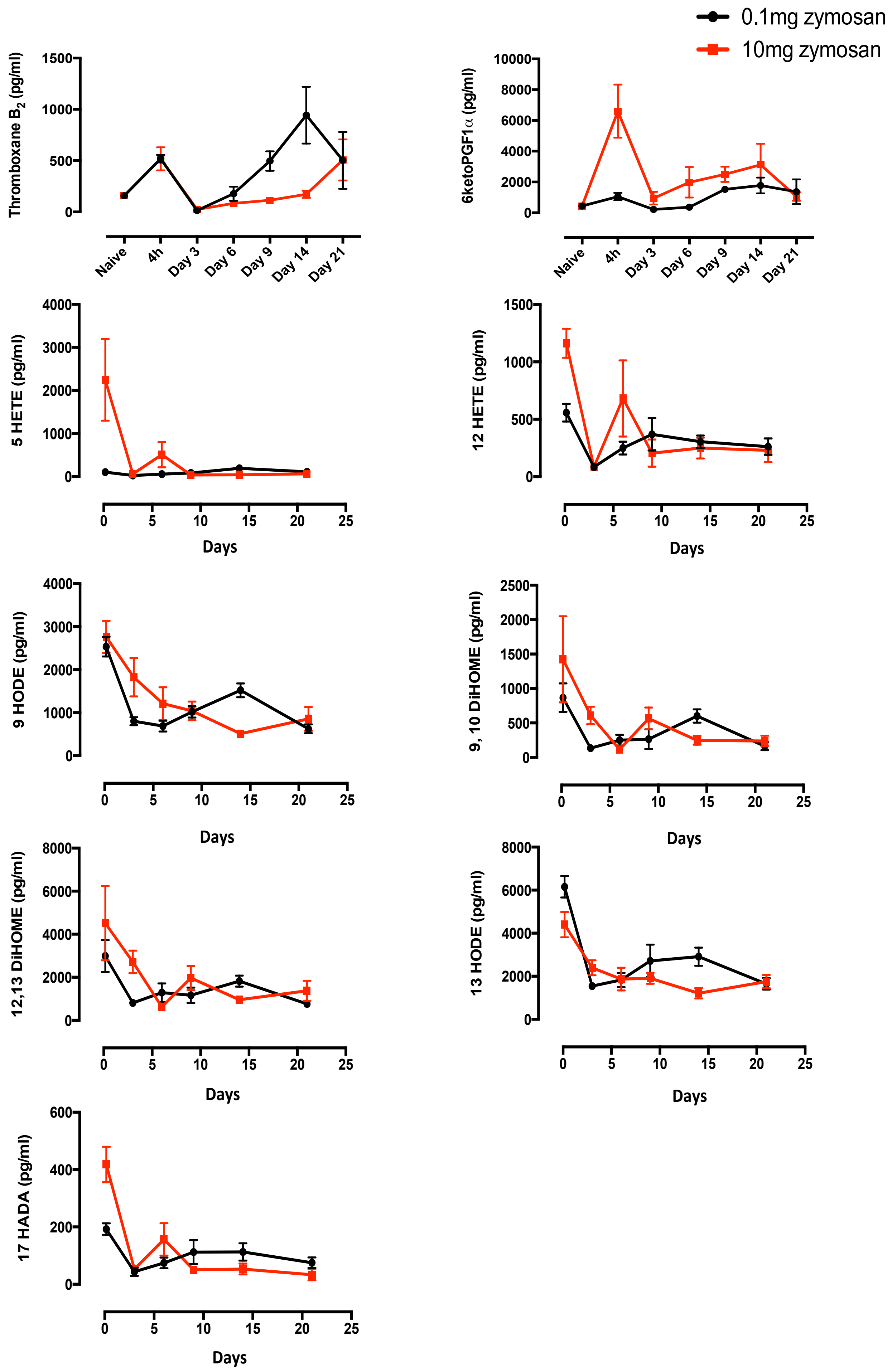


**Figure S1.** Lipidomic profiling of cell-free inflammatory exudate from mice that received either 0.1 or 10mg zymosan i.p. Related to Figure 2

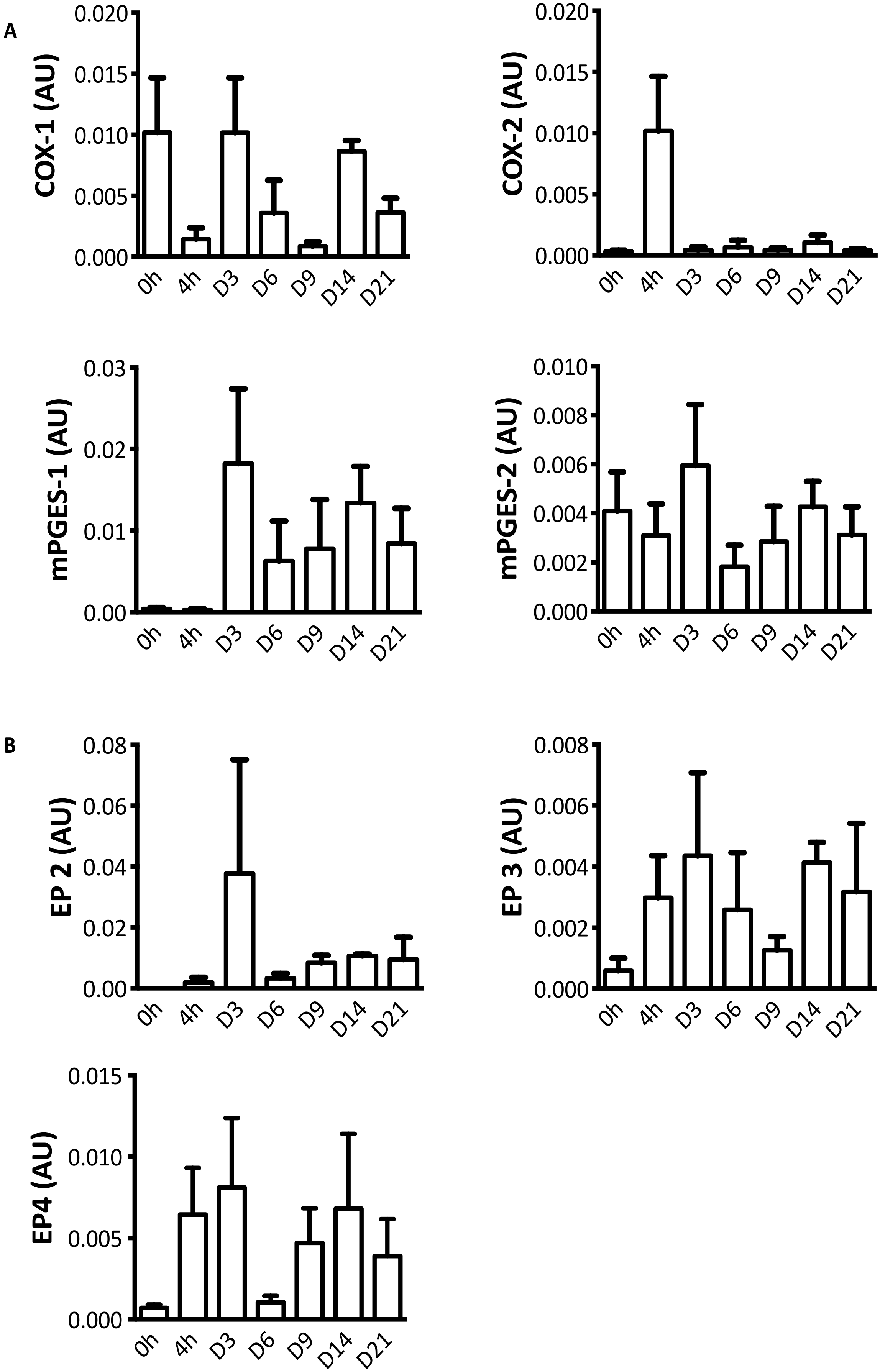


**Figure S2.** (A) Densitometry values for the temporal expression of COX and mPGES isoforms and (B) $PGE_2$ receptors on bulk cells from a mouse peritonitis driven by 0.1mg zymosan i.p. Related to Figures 2 and 3.

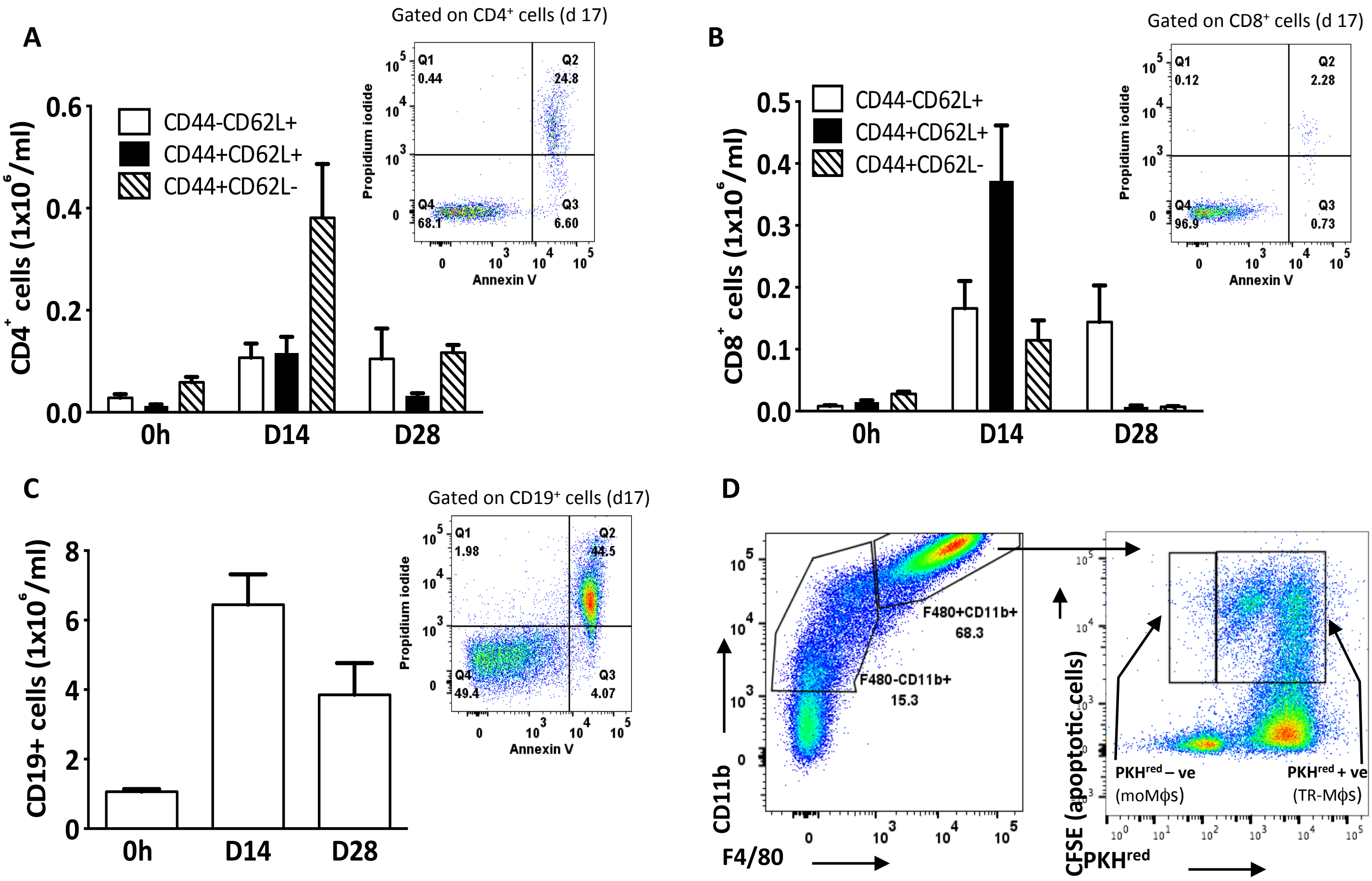


**Figure S3.** Expansion and contraction of **(A-B)** memory T cell populations as well as **(C)** B cells from day 14 post 0.1mg zymosan injection. The reduction in lymphocyte numbers was due, at least in part, to programmed cell death with apoptotic bodies **(insert panels to A-C)** being cleared by **(D)** tissue-resident macrophages. This assertion is derived from an experiment where we injected apoptotic lymphocytes labelled with $PKH^{red}$ into the peritoneum at day 14 finding that $PKH^{red}$ positive tissue-resident macrophages preferentially phagocytosed these cells and not infiltrating monocyte-derived macrophages. Related to Figures 6 and 7

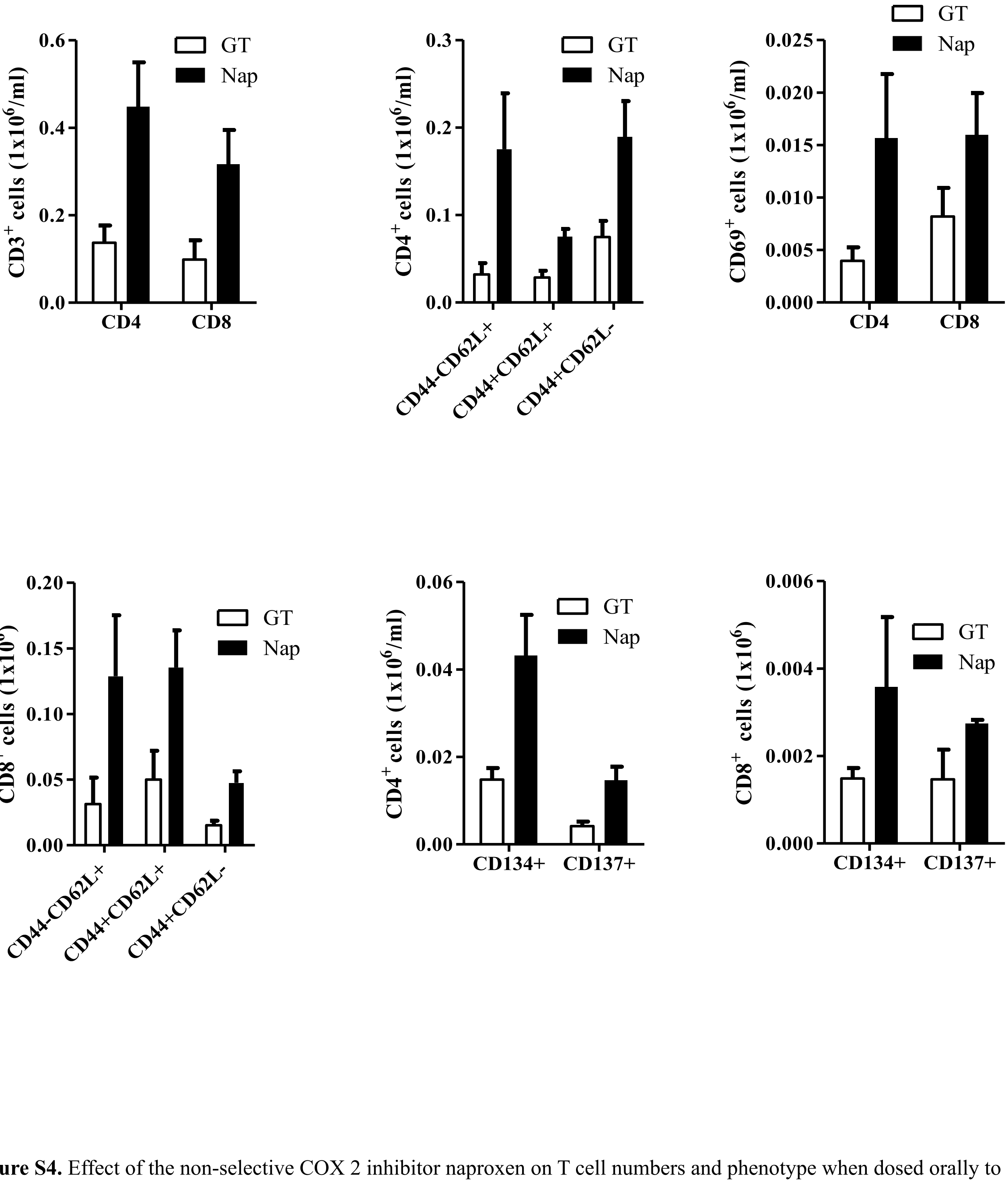


**Figure S4.** Effect of the non-selective COX 2 inhibitor naproxen on T cell numbers and phenotype when dosed orally to mice bearing a 0.1mg zymosan-induced peritonitis from day for two weeks. Related to Figure 6.
