# Supplementary material for: Inflammatory Resolution Triggers a Prolonged Phase of Immune Suppression through COX-1/mPGES-1-Derived Prostaglandin E2
Source: Cell Rep. 2017 Sep 26;20(13):3162–75. doi: 10.1016/j.celrep.2017.08.098 (PMC5639146; doi:10.1016/j.celrep.2017.08.098)
Supplement: Document S2. Article plus Supplemental Information [file mmc2.pdf]

Cell Reports

Article

Inflammatory Resolution Triggers a Prolonged Phase of Immune Suppression through COX-1/mPGES-1-Derived Prostaglandin E_2

Graphical Abstract

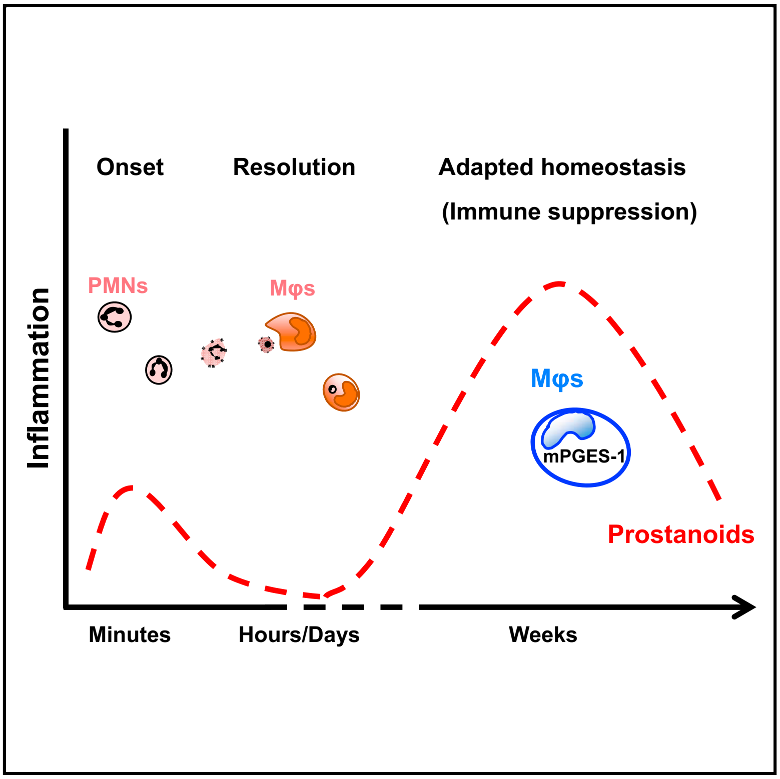

Highlights

- Inflammatory resolution triggers T/NK cell infiltration, which synthesizes IFNγ
- Through IP-10, IFNγ indirectly triggers monocyte-derived macrophage infiltration
- Macrophages are directly acted upon by IFNγ to make abundant PGE_2
- PGE_2 exerts a phase of post-inflammation immune suppression and tolerance

Authors

Justine Newson, Madhur P. Motwani, Alexandra C. Kendall, ..., Sarah James, Roel P.H. De Maeyer, Derek W. Gilroy

Correspondence

d.gilroy@ucl.ac.uk

In Brief

Inflammatory resolution was believed to lead affected tissues back to homeostasis. Newson et al. now find that resolution triggers a prolonged phase of localized immune suppression called "adapted homeostasis." This phase is mediated by macrophage-derived prostaglandin E_2 derived from COX-1/mPGES1 and is crucial in preventing the development of autoimmunity.

CrossMark

Newson et al., 2017, Cell Reports *20*, 3162–3175
September 26, 2017 © 2017 The Authors.
http://dx.doi.org/10.1016/j.celrep.2017.08.098

CellPress

OPEN ACCESS
CellPress

Cell Reports
Article

Inflammatory Resolution Triggers a Prolonged Phase of Immune Suppression through COX-1/mPGES-1-Derived Prostaglandin E_2

Justine Newson,[1] Madhur P. Motwani,[1] Alexandra C. Kendall,[2] Anna Nicolaou,[2] Giulio G. Muccioli,[3] Mireille Alhouayek,[3] Melanie Bennett,[1] Rachel Van De Merwe,[1] Sarah James,[1] Roel P.H. De Maeyer,[1] and Derek W. Gilroy[1,4,*]
[1]Centre for Clinical Pharmacology and Therapeutics, Division of Medicine, 5 University Street, University College London, London WC1E 6JJ, UK
[2]Division of Pharmacy and Optometry, School of Health Sciences, Faculty of Biology, Medicine and Health, The University of Manchester, Stopford Building, Oxford Road, Manchester M13 9PT, UK
[3]Bioanalysis and Pharmacology of Bioactive Lipids Research Group, Louvain Drug Research Institute, Université Catholique de Louvain, Av. E. Mounier, 72 (B1.72.01), 1200 Bruxelles, Belgium
[4]Lead Contact
*Correspondence: d.gilroy@ucl.ac.uk
http://dx.doi.org/10.1016/j.celrep.2017.08.098

SUMMARY

Acute inflammation is characterized by granulocyte infiltration followed by efferocytosing mononuclear phagocytes, which pave the way for inflammatory resolution. Until now, it was believed that resolution then leads back to homeostasis, the physiological state tissues experience before inflammation occurred. However, we discovered that resolution triggered a prolonged phase of immune suppression mediated by prostanoids. Specifically, once inflammation was switched off, natural killer cells, secreting interferon γ (IFNγ), infiltrated the post-inflamed site. IFNγ upregulated microsomal prostaglandin E synthase-1 (mPGES-1) alongside cyclo-oxygenase (COX-1) within macrophage populations, resulting in sustained prostaglandin (PG)E_2 biosynthesis. Whereas PGE_2 suppressed local innate immunity to bacterial infection, it also inhibited lymphocyte function and generated myeloid-derived suppressor cells, the net effect of which was impaired uptake/presentation of exogenous antigens. Therefore, we have defined a sequence of post-resolution events that dampens the propensity to develop autoimmune responses to endogenous antigens at the cost of local tissue infection.

INTRODUCTION

Acute inflammation is a protective reaction of the microcirculation initiated after infection and/or injury with the aim of eliminating the inciting stimulus while promoting tissue repair and healing (Lawrence et al., 2002; Nathan, 2002). Once the injurious agent has been eliminated, a well-described sequence of events called resolution ensues. These include pathogen clearance (Segal and Peters, 1976), deactivation of pro-inflammatory signaling pathways (Stoecklin and Anderson, 2006), catabolism of cytokines and chemokines (Jamieson et al., 2005), as well as inhibition of granulocyte recruitment (Rajakariar et al., 2007). Thereafter, the infiltrated granulocytes die by apoptosis and are cleared by tissue-resident macrophages (Savill et al., 1989). This entire process is relatively rapid, occurring within 3–5 days.

Upon successful resolution, there is the view that the inflamed tissue reverts to the cellular and biochemical state it experienced before infection/injury. However, there is increasing evidence that resolution is not the end of innate immune-mediated responses to infection but that cellular and biochemical events triggered by the resolution cascade influence subsequent adaptive immune responses (León et al., 2007; Nakano et al., 2009; Newson et al., 2014; Wakim and Bevan, 2011). There is also the emerging view that some infections cause "immunological scarring" such that, despite effective clearance of the inciting stimulus, rather than reverting to homeostatic normality, chronic inflammation develops (Fonseca et al., 2015; Kuperman et al., 2002). Taken together, these investigations suggest that resolution, as we currently understand it, is not the end of innate immune-mediated responses to infection. Instead, once the cardinal signs of inflammation have abated, there is a great deal of immunological activity occurring at the sub-clinical level, at the site of inflammation, which dictates the long-term physiological fate of tissues post-injury.

In support of this emerging concept, we found that, following resolution of acute peritonitis, there was the sustained infiltration of myeloid and lymphoid cells into the peritoneum that persisted for months (Newson et al., 2014). We hypothesized that this post-resolution infiltrate bridged the gap between innate and adaptive immunity as depleting myeloid cells, for instance, during this phase blunted lymph node expansion. Moreover, a population of these infiltrated myeloid cells was retained in the peritoneum long term and dictated the severity and longevity of subsequent innate immune-mediated responses to secondary inflammatory stimuli (Newson et al., 2014; Yona et al., 2013). Following on from this, we have now observed a prolonged phase of prostanoid biosynthesis, namely PGE_2,

 © 2017 The Authors.
This is an open access article under the CC BY license (http://creativecommons.org/licenses/by/4.0/).

occurring within a few days of acute inflammation resolving. In our attempts to understand what triggered PGE_2 and decipher its role in post-resolution biology, we found robust cyclo-oxygenase (COX-1)/PGES-1 expression in myeloid cells that was triggered by interferon γ (IFNγ). It transpires that post-resolution PGE_2 is potently immune suppressive during this phase, with a role in maintaining immune tolerance, but at the cost of increased susceptibility to secondary infection.

RESULTS

IFNγ-Induced IP-10/CXCL10 Triggers Post-resolution Monocyte Infiltration

Resolution of acute inflammation in response to 0.1 mg zymosan occurs within 72–96 hr (Newson et al., 2014). Starting at day three post-zymosan, and coincident with the end of resolution, we noted the infiltration of natural killer (NK) cells peaking in number at days 9–14 and declining thereafter (Figure 1A); an equivalent trend in this model was also seen with CD4 and CD8 T cells (Newson et al., 2014). Mirroring NK cells, as well as T cells (Newson et al., 2014), was an increase in cell-free inflammatory exudate IFNγ as well as monokine induced by gamma IFN (MIG/CXCL9) and IFNγ-induced protein 10 (IP-10/CXCL10) (Figures 1B–1D, respectively), with IFN-γ being secreted by NK cells as well as CD4 and CD8 T cells (Figures 1E and 1F). Given the relative paucity of the classic monocyte chemoattractant MCP-1 during this post-resolution phase (Figure 1G), we questioned whether IP-10, which is also a monocyte chemoattractant (Taub et al., 1993), was responsible for post-resolution monocyte accumulation in the peritoneum.

We injected zymosan into MIIG (macrophages insensitive to IFNγ) mice. These mice express a CD68-restricted dominant-negative IFNγ receptor that renders $CD68^+$ macrophages insensitive to IFNγ (Lykens et al., 2010). We found substantially reduced numbers of monocytes at day 14 in these animals compared to wild-type controls (Figure 1H). To prove that the infiltration of monocytes was caused by IP-10, we injected wild-type mice bearing 0.1-mg-zymosan-induced peritonitis with blocking antibodies to IP-10, MIG, or MCP-1. It transpires that blocking only IP-10 reduced monocyte numbers during post-resolution (representative data at day 14; Figure 1I). Therefore, the infiltration of monocytes into post-resolving tissue is caused by IP-10, most likely triggered by T-cell- and NK-cell-derived IFNγ.

Elevated and Sustained Post-resolution Prostanoid Biosynthesis

Liquid chromatography-tandem mass spectrometry (LC-MS/MS) analysis of cell-free inflammatory exudates revealed a peak in PGE_2 at day 14 post-0.1 mg zymosan being four times higher than levels seen within the first few hours of inflammatory onset (Figure 2A). A similar profile was seen with thromboxane (Tx)B_2 and prostacyclin (PGI_2; measured as 6-keto $PGF_{1\alpha}$), but not lipoxygenase or cytochrome p450 metabolites (Figure S1).

Western blotting analysis of total cells from the peritoneum showed that COX-1 was expressed in cells of the naive cavity, with levels declining during acute inflammation (~4 hr) but rising again from day 3 (Figure 2B). In contrast, COX-2 was absent from the naive peritoneum, transiently increased during early onset (4 hr) and disappeared thereafter. Alongside changes in COX-1 expression were increases in both microsomal prostaglandin E synthase-1 (mPGES-1) and -2 isoforms to levels persistently higher than those seen in the naive cavity (Figure 2B; densitometry values are shown in Figure S2A). These data suggest that post-resolution increases in levels of PGE_2 were not derived from COX-2, as might be expected, but from COX-1 coupled with mPGES isoforms.

Analysis of monocytes and macrophages up to day 28 revealed at least three populations, namely $Ly6C^{hi}/F4\text{-}80^-$ and $Ly6C^{lo}/F4\text{-}80^-$ monocytes as well as $F4\text{-}80^{hi}/CD11b^+/MHC\text{-}II^{hi}$ macrophages (data for day 14 are shown in Figure 2C). Further analysis of the $F4\text{-}80^{hi}/CD11b^+/MHC\text{-}II^{hi}$ macrophage population using PKH^{red} cell-tracking experiments revealed that approximately 80% comprised macrophages that were resident to the naive cavity (before zymosan injection), with the remaining cells being monocyte derived (Figure 2D). Fluorescence-activated cell sorting (FACS) these respective populations followed by quantitative real-time PCR traced the expression of COXs and their downstream synthases to both tissue-resident as well as infiltrating monocyte-derived macrophages (Figure 2E). Given these expression profiles, it would appear that COX-1 and inducible mPGES-1 are the predominate source of post-resolution PGE_2 expressed within myeloid cells; their expression at RNA level was not detectable in lymphoid cells at this time point (data not included).

Post-resolution EP Receptor Expression

It transpires that PGE_2 receptor (EP)1 was not detectable on total cells at the protein level (data not shown) whereas EP2–4 were found throughout inflammation, resolution, and post-resolution phases (Figure 3A; densitometry values are shown in Figure S2B). We next FACS sorted T and B cell populations from the naive or post-resolution cavity as well as various mononuclear phagocytes (monocytes, monocyte-derived macrophages, as well as tissue-resident macrophages) to determine cellular expression of EP receptors. At the message level, EP2 and EP4 were the most abundantly expressed by resident macrophages as well as monocyte-derived macrophages (Figure 3B); T and B cells also expressed these receptors (Figure 3C). Data from experiments in Figures 6A and 6B reveal EP4 to be most functionally important on lymphocytes.

Collectively, these data reveal an unprecedented increase and persistent temporal profile of prostanoid synthesis after inflammation has resolved, driven by COX-1/mPGES with receptors for these lipids expressed on cells of the post-inflamed cavity.

IFNγ Drives Inducible mPGES-1 Expression

Given that the profile of IFNγ in this model preceded that of PGE_2 and that IFNγ has been shown to trigger mPGES in colonic epithelial cells (Wright et al., 2004), we investigated whether IFNγ was responsible for post-resolution prostanoid synthesis. Incubating peritoneal macrophages with this cytokine (as well as IP-10 and MIG at concentration found in the cavity at day 9) resulted in an increase in mPGES-1 with no effect seen on mPGES-2 levels (Figures 4A and 4B). Taking this further, we injected zymosan into MIIG mice and, at day 14, FACS sorted

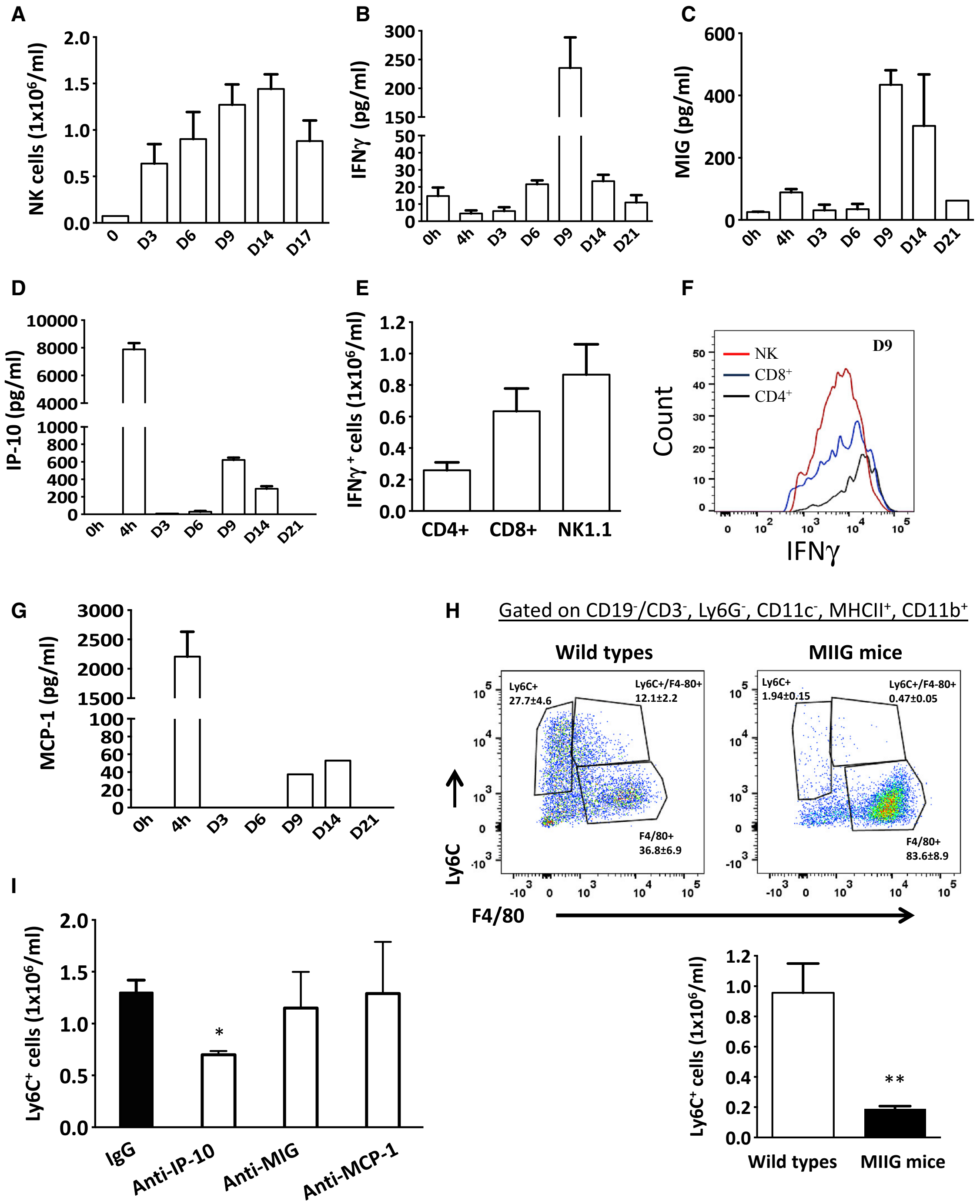

Figure 1. IFNγ-Induced IP-10/CXCL10 Triggers Post-resolution Monocyte Infiltration

Wild-type mice had 0.1 mg zymosan injected into their peritoneal cavity with the cellular infiltrate analyzed by polychromatic flow cytometry starting from when inflammation typically resolves in this model.

(A–D and G) Data show the accumulation of (A) NK cells followed by cell-free inflammatory exudates levels of (B) IFNγ, (C) MIG, (D) IP-10, and (G) MCP-1.

(E and F) The key post-resolution cell types expressing IFN (E), and the intracellular staining for IFNγ in these cells at days 9/14 after zymosan injection (F).

(H and I) Panels in (H) show the numbers of monocytes in MIIG mice (animals whose macrophages are insensitive to IFNγ) at day 14 after zymosan, whereas (I) confirms that IP-10 only is responsible for the infiltration of post-resolution monocytes in this model. *$p \leq 0.05$; **$p \leq 0.01$.

Data are expressed as mean ± SEM; n = 5 mice/group.

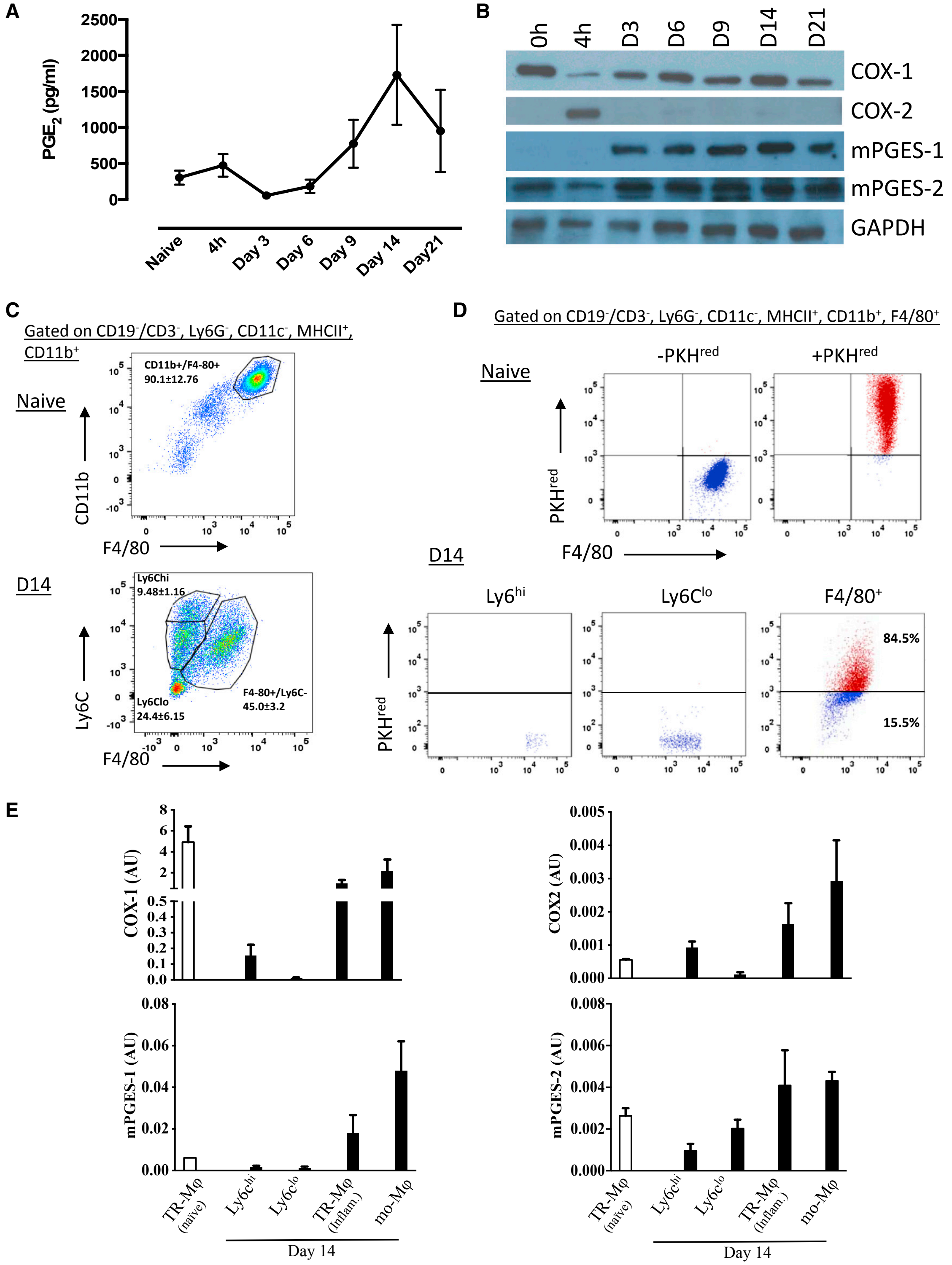

Figure 2. Lipidomic Profiling of Inflamed and Post-resolved Tissues

(A) Peritoneal cell-free inflammatory exudates from mice that received 0.1 mg zymosan were analyzed by LC-MS/MS at indicated time points.

(B) Total peritoneal cells were subjected to western blotting to determine the temporal expression of the prostanoid-generating enzyme cascade.

(C) The profile of mononuclear phagocytes in the naive and post-inflamed cavity.

(legend continued on next page)

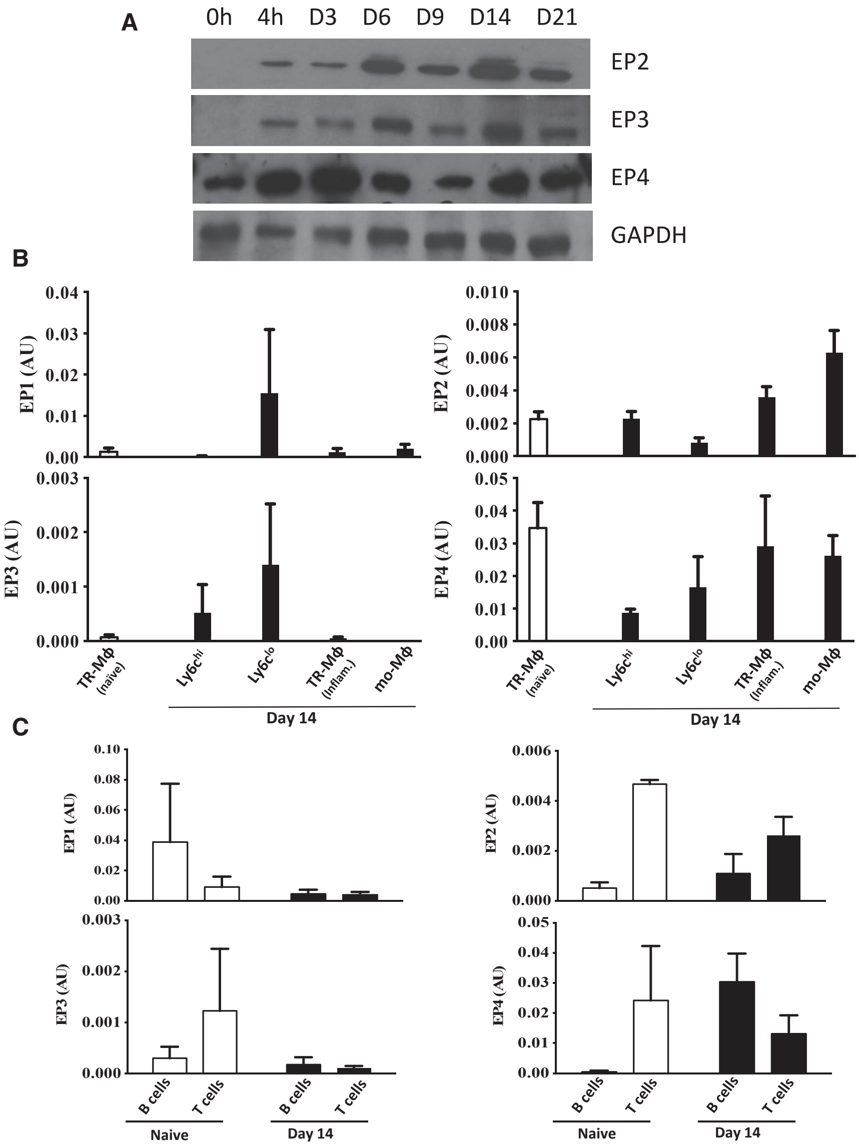

Figure 3. Post-resolution EP Receptor Expression

(A) Total peritoneal cells were subjected to western blotting to determine the temporal expression of prostanoid receptors.

(B and C) In addition to (B) monocyte/macrophage populations, the post-resolution infiltration of (C) $CD4^+$, $CD8^+$, and $CD19^+$ lymphocytes were FACS sorted to determine EP expression levels on individual post-resolution myeloid and lymphoid populations.

Data are presented as mean ± SEM; n = 6 mice per group.

post-resolution macrophage populations and subjected them to qPCR, revealing a substantial decrease in mPGES-1, but not mPGES-2 (Figures 4C and 4D). We also found that PGE_2, when incubated with post-resolution T cells, inhibited their secretion of IFNγ in an EP4-dependent manner (Figure 4E). It was therefore not surprising to see an increase in IFNγ as well as MIG and IP-10 at day 21 in mice treated with an EP4 receptor antagonist from days 6 to 21 post-zymosan (Figures 4F–4H). These data show that type II IFN triggers mPGES-1 expression and is responsible for post-resolution prostanoid biosynthesis, with PGE_2 acting as a negative feedback inhibitor of IFNγ synthesis.

Post-resolution PGE_2: A Role in Innate Immune Suppression

As PGE_2 is a potent suppressor of innate immunity (O'Brien et al., 2014; Serezani et al., 2007), we injected S. pneumoniae 21 days after 0.1 mg zymosan and noted that these mice became noticeably sicker compared to naive controls that received an equivalent amount of bacteria, with their degree of clinical illness becoming progressively worse up to 72 hr, when these mice had to be euthanized. Importantly, inhibiting PGE_2 synthesis or antagonizing its EP4 receptor reversed animal sickness and resulted in greater clearance of bacteria (Figures 5A and 5B, respectively).

Post-resolution PGE_2 Inhibits Adaptive Immunity

In addition to innate immunity, PGE_2 also has potent modulatory effects on adaptive immunity (Kalinski, 2012). Coincident with the second peak in PGE_2 at day 14 in this 0.1 mg zymosan model was a reduction in numbers of memory T and B cells, with their contraction due, at least in part, to programmed cell death, with apoptotic bodies being cleared by tissue-resident macrophages (Figure S3) in line with that reported previously (Newson et al., 2014; Uderhardt et al., 2012).

Next, we found that PGE_2 inhibited the ex vivo proliferation of T and B cells sorted from the peritoneum 14 days post-zymosan in an EP4-dependent manner (Figures 6A and 6B). Accordingly, dosing animals from day 6 to day 21 post-zymosan with the selective EP4 receptor antagonist MF-498 resulted in an increase in numbers of peritoneal $CD3^+$ T cells (Figure 6C); equivalent data were obtained with the non-selective COX inhibitor

(D) The relative proportions of tissue-resident macrophages, which were labeled positively with PkH-PCL^{red} when injected into the naive peritoneum versus infiltrating monocyte-derived macrophages, which are cell tracker negative.

(E) FACS was used to separate tissue-resident macrophages (TR-Mϕ^{naive}), tissue-resident macrophages that experience inflammation (TR-M$\phi^{inflam.}$), infiltrated $Ly6c^{hi/lo}$ monocytes, and infiltrating monocyte-derived macrophages (mo-Mϕ) to determine cell expression of COXs and their downstream synthase.

Data are expressed as mean ± SEM; n = 5 mice/group.

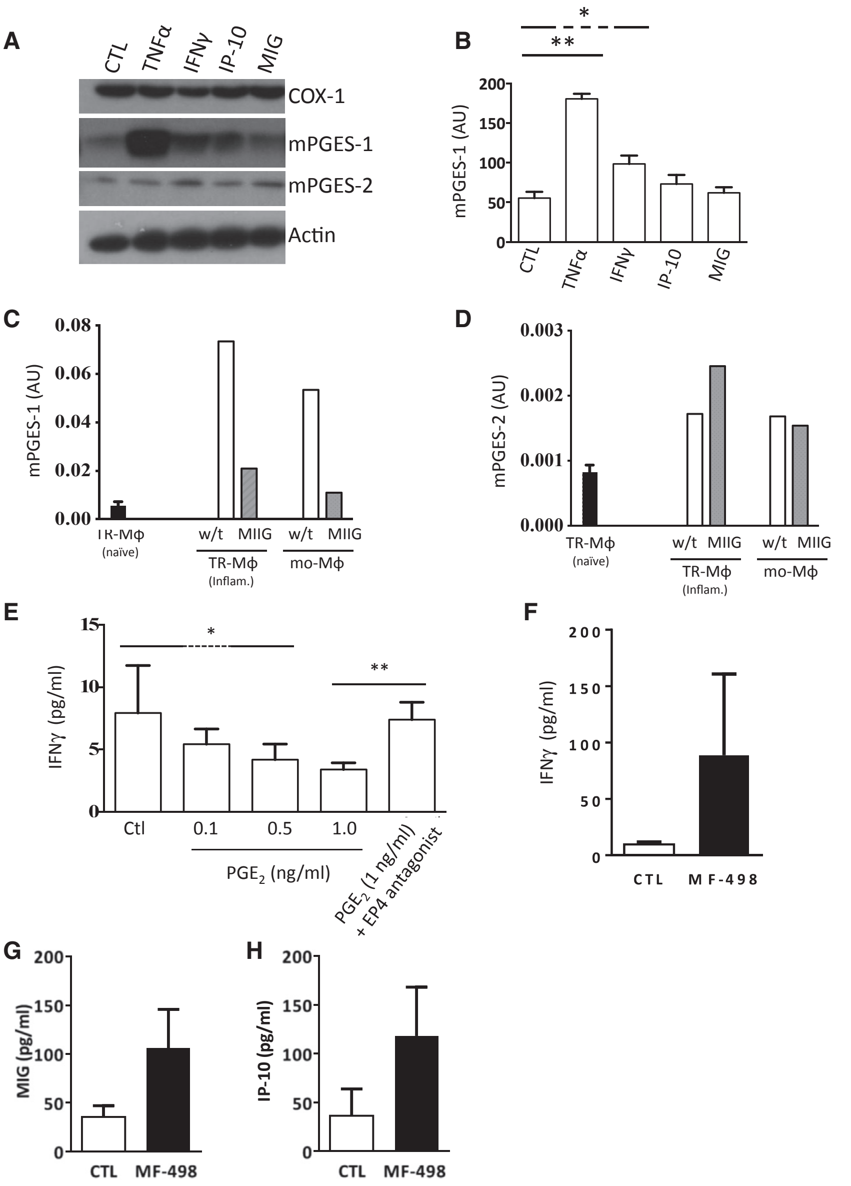

Figure 4. IFNγ Triggers Post-resolution mPGES-1 Expression

(A and B) The effects of IFNγ as well as factors it triggers, including MIG and IP-10 (used at levels found at day 9 in the peritoneum), on macrophage expression of COX and its downstream synthase isoform expression (A), and (B) shows quantification for mPGES-1.

(C and D) Zymosan was injected into macrophages insensitive to IFNγ (MIIG) mice, from which post-resolution macrophage populations were FACS sorted followed by qPCR to determine (C) mPGES-1 and (D) mPGES-2 expression levels.

(E–H) T cells were isolated from the peritoneum at day 14 post-zymosan and incubated ex vivo with PGE_2 (E), while the effects of an EP4 agonist on cell-free exudate levels of IFNγ (F), MIG (G), and IP-10 (H) was determined at day 21.

$^{*}p \leq 0.05$; $^{**}p \leq 0.01$. Data are expressed as mean ± SEM; n = 5 mice/group.

naproxen (Figure S4). Blocking EP4 also skewed $CD4^+/CD44^+/CD62L^-$ memory T cells toward a Th1 phenotype based upon an increased release of IFNγ from these cells (Figure 6D). Another important observation following the inhibition of post-resolution PGE_2 was a decrease in numbers of myeloid-derived suppressor cells (Figure 6E), coincident with an increase in peritoneal dendritic cell numbers (Figure 6F), a differential effect that is well-described in the literature (Obermajer and Kalinski, 2012; Obermajer et al., 2011). The phenotype and suppressive function of post-resolution myeloid-derived suppressor cells in panel C of Figure 6E were identified as reported previously (Newson et al., 2014).

From these data, we predict that PGE_2 is highly immune suppressive during the post-resolution phase of acute inflammatory responses. To test this hypothesis, we injected methylated BSA into the cavity of mice bearing a 0.1-mg-zymosan-induced peritonitis at day 14 and found that a very weak immune response was raised to this antigen compared to when mBSA was injected into naive mice; this immune suppression was rescued by COX inhibition (Figure 6G).

mPGES-1/PGE2 Is Absent in Inflammation Triggered by 10 mg Zymosan

Injecting higher levels of the same stimulus (10 mg zymosan intraperitoneally [i.p.]) caused a pronounced local granulocytic infiltrate and systemic cytokine storm (Newson et al., 2014). Nonetheless, this inflammatory response also resolved such that, within days, the composition of the peritoneum in these mice was similar to that of mice that received 0.1 mg zymosan in terms of neutrophil and pro-inflammatory cytokine profiles, classical determinants of resolution (Newson et al., 2014). Indeed, in response to 10 mg zymosan, monocytes and macrophages were also detected in the cavity up to day 21 with proportionally more $Ly6C^{lo}/F4\text{-}80^-$ monocytes observed (Figure 7A) compared to inflammation triggered by 0.1 mg zymosan (see Figures 2C and 2D; Newson et al., 2014). Importantly, of the $F4\text{-}80^{hi}/CD11b^+/MHC\text{-}II^{hi}$ mature macrophage populations, the majority were derived from monocytes, with tissue-resident macrophages representing on average ~5% of the mononuclear phagocyte population (Figure 7B).

In addition, both IFNγ and IP-10 were undetectable throughout the response to 10 mg zymosan; instead, monocyte chemotactic protein-1 (CCL2; Figure 7C) was followed by the infiltration of the monocyte-derived macrophages bearing a

A

Murine sickness score						
	0h	24h	48h	60h	72h	Cumulative
Naïve + *St. pneu*	0	0	1	0	0	**1**
Day 21 + *St. pneu*	0	0	2.3	1.7	2.0	**7**
Day 21+ *St. pneu + MF-498*	0	0	1	0	0	**1**
Day 21 + *St. pneu + naproxen*	0	0	1	1	1	**2**
Post res. 10mg zymo. + St. pneu	0	1	0	0	1	**2**

B

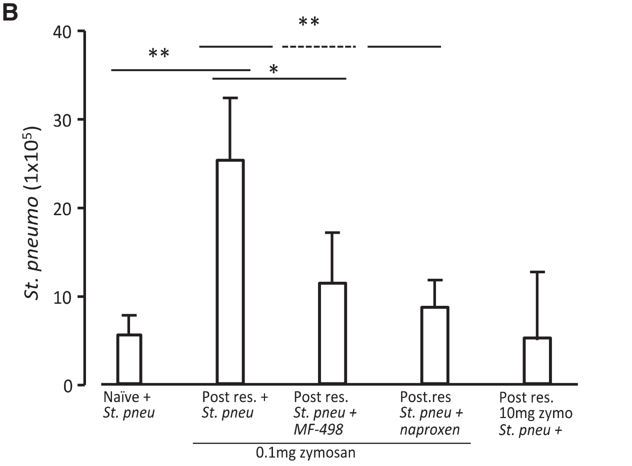

Figure 5. Post-resolution Tissues Are in a State of PGE_2-Mediated Innate Immune Suppression

Live bacteria (*St. pneumonia*) were injected into either naive mice or mice bearing a 0.1-mg-zymosan-induced peritonitis at day 21. Separate groups of zymosan-injected mice were dosed from day 6 post-zymosan injection with either MF-498 (EP4 antagonist) or naproxen for two weeks.

(A) How mice over time became progressively sick following bacteria. This was assessed using a "murine sickness score," which was developed in association with the UCL animal welfare group and veterinary surgeon; see Experimental Procedures.

(B) The number of surviving bacteria in the blood of these animals. $^{*}p \leq 0.05$; $^{**}p \leq 0.01$.

Data are expressed as mean ± SEM; n = 6 mice/group.

largely pro-inflammatory phenotype in comparison to equivalent populations triggered by 0.1 mg zymosan (Figure 7D). In addition, the relatively few tissue-resident macrophages recovered from the 10 mg zymosan model expressed less TIM4, ALOX15, and transforming growth factor β1 (TGF-β1) (markers of efferocytosis) compared to equivalent counterparts recovered from 0.1 mg zymosan (Figure 7D). The lack of these dedicated efferocytosing phagocytes in the 10 mg model was associated with the accumulation of secondary apoptotic lymphocytes and granulocytes bearing higher nucleic acid stain and annexin V labeling (Figure 7E) compared to apoptosing lymphocytes seen following 0.1 mg zymosan (Figure S3). Not surprisingly, we detected antibodies to double-stranded DNA (dsDNA) in the serum of these animals rising from day 21 post-10 mg zymosan (Figure 7F). Finally, exudates of mice injected with 10 mg zymosan revealed no increased PGE_2 levels or the expression of mPGES-1 (Figure 7G). We therefore investigated the impact of exogenously adding a stable PGE_2 analog to mice bearing a 10-mg-zymosan-induced inflammation and found that dosing daily from day 6 up to day 21 resulted in a reduction in serum antibodies to dsDNA (Figure 7H).

DISCUSSION

Whereas the origins of chronic inflammatory or autoimmune diseases remain unclear, multiple factors have been implicated, including genetics, age, and environmental signals. Pathogens are the main environmental factors postulated to drive autoimmunity, with several hypotheses proposed to explain their mechanism of action, including molecular mimicry and bystander activation (Fujinami et al., 1983, 2006; Woodland and Blackman, 1992). In addition, persistence of the infection arising from a defective innate immune system (Dinauer, 1993; Morgenstern et al., 1997; Segal, 1996) or failure to engage adaptive immunity (Teijaro et al., 2013; Wilson et al., 2013) can also lead to chronic inflammation and autoimmunity.

There is also evidence of immune dysfunction leading to chronic disease occurring long after clearance of the infectious stimulus. For instance, in a murine model of Sendai-induced para-influenza, despite clearing the infection, mice progressed to develop an asthma-like disease mediated by sustained activity of NK T cells driving macrophages to produce interleukin-13 (IL-13) (Kuperman et al., 2002). More recently, mice that received a single inoculum of *Yersinia pseudotuberculosis* experienced immune disruption in the gut weeks after bacterial clearance (Fonseca et al., 2015). This disruption was characterized by lymphatic leakage in the mesenteric adipose tissue that redirected dendritic cells to the adipose compartment, thereby preventing their proper accumulation in the mesenteric lymph node. Consequently, mucosal immune functions, including tolerance and protective immunity, were persistently compromised.

Thus, even if the inciting stimulus is cleared, there is evidence of local "immunological mal-adaption," predisposing tissues to chronic inflammation occurring months or years after the initial exposure, at least in response to some infections. However, the nature of this post-inflammation, immune mal-adaptation is not clearly understood, and further research is warranted in this area.

In this paper, we found that, following the resolution of acute inflammation triggered by low-dose zymosan, there is a prolonged sequence of events at the cellular and molecular level and occurring in a sub-clinical manner that may prevent the development of some autoimmune diseases. One of the key events in this process is the sustained synthesis of PGE_2, which

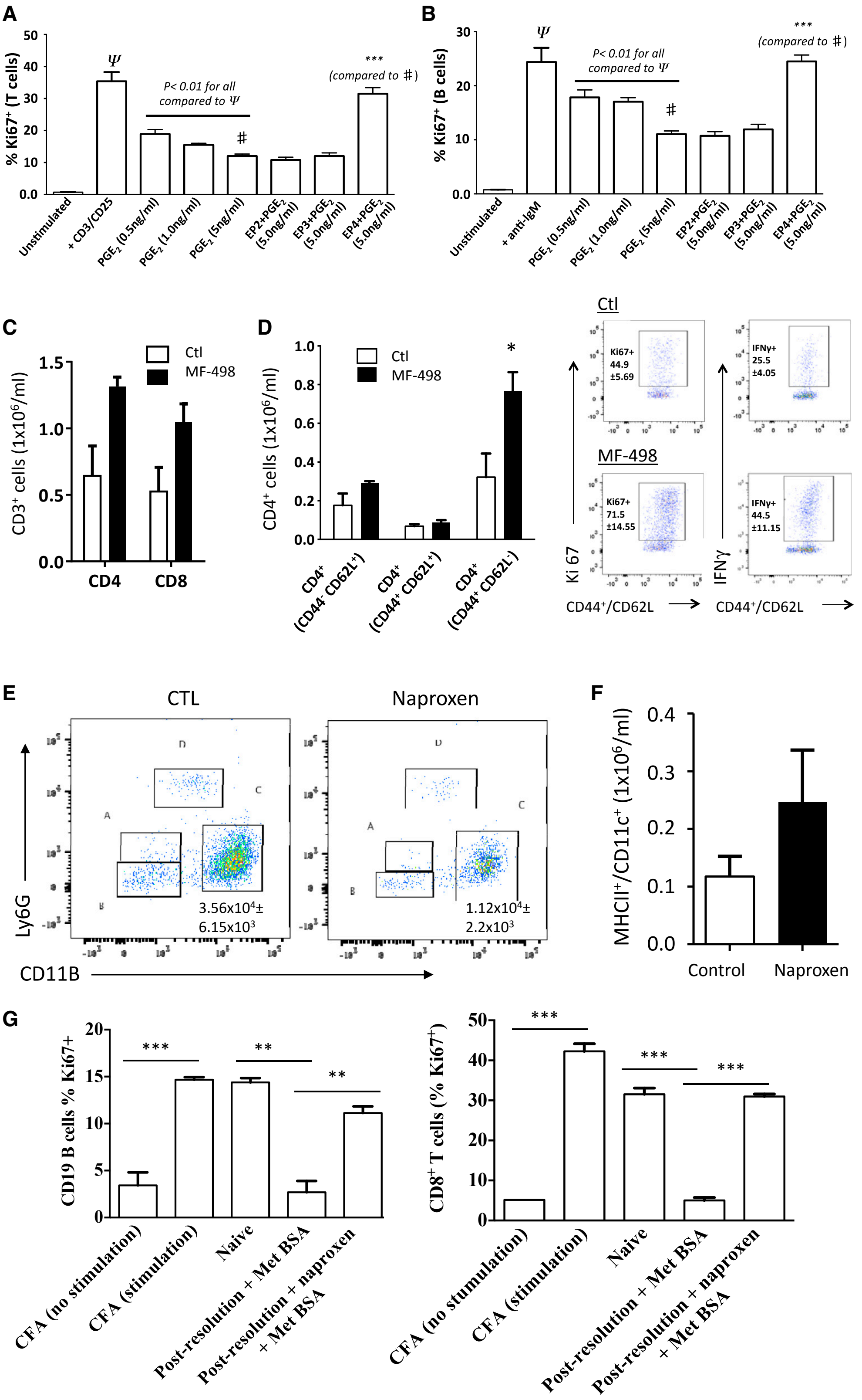

(legend on next page)

is derived from macrophage COX-1/mPGES and that is triggered by IFNγ. It transpires that this post-resolution phase of prostanoid biosynthesis creates a window of susceptibility to infection on the one hand, while also impairing the host's ability to generate adaptive immune response to antigens on the other. We interpret these data as an evolutionary trade-off, where the threat of localized infection is more desirable than the specter of developing autoimmunity to an endogenous antigen, such as those generated by apoptotic cells during resolution, or by citrullinated protein or collagen fragments following acute inflammation.

The use of 10 mg zymosan in these studies has been somewhat serendipitous in that, whereas inflammation did eventually resolve, at least as defined by polymorphonuclear neutrophil (PMN) clearance and a return of total peritoneal cells to numbers similar to the pre-inflamed cavity (Newson et al., 2014), the post-resolution 10 mg zymosan peritoneum did not trigger NK cell infiltration or elaborate IFNγ/IP-10. Moreover, the numbers of tissue-resident macrophages recovered from the cavity of these animals was considerably less than that seen post-0.1 mg zymosan whereas their phenotype was suggestive of a diminished capacity to efferocytose apoptotic cells. Instead, the post-resolution 10 mg cavity saw the infiltration of monocyte-derived macrophages bearing an M1-like phenotype not expressing COX-1/mPGES or synthesizing prostanoids. We propose that the lack of efferocytosing tissue-resident macrophages resulted in the accumulation of secondary apoptotic PMNs and lymphocytes, which, in the absence of immune-suppressive PGE_2, ultimately leads to the accumulation of antibodies to dsDNA. Whereas these effects were inhibited when a stable analog of PGE_2 was dosed to 10 mg zymosan mice from days 8 to 21, the precise mechanisms underlying these findings are not known and beyond the scope of this paper but most likely arise from the inhibition of memory T cell and/or B cell proliferation or generation of suppressor myeloid or T cells.

Consequently, data presented here, as well as that published by others (Fonseca et al., 2015; Kuperman et al., 2002), calls for a clearer definition of inflammatory resolution. Until now, there was the view that homeostasis is restored once acute inflammation resolves (Serhan et al., 2007). In other words, inflamed tissues revert to the state they experienced before infection/injury. Arising from the above, it is clear that this is not the case. This prompts us to put forward a revised definition of resolution. The first occurs following transient inflammation and triggers a sequence of events resulting in "resolution leading to adapted homeostasis". This is the desired outcome of innate immune-mediated responses to infection/injury, resulting in the maintenance of tolerance and prevention of chronic inflammation. The second is where events leading to "adapted homeostasis" are dysregulated by the inflammatory stimulus and/or are inherently absent/disrupted in the host. For this, we propose the term "resolution leading to mal-adapted homeostasis". This is the undesired outcome, which we suspect underpins the etiology of at least some chronic inflammatory and autoimmune diseases.

The consensus is that IFNγ is essentially pro-inflammatory in nature. However, there is evidence to suggest that it may also play a beneficial role in controlling autoimmunity and chronic inflammatory diseases. For instance, mice with a disrupted IFNγ gene are susceptible to experimental autoimmune encephalomyelitis (Ferber et al., 1996) whereas collagen-induced arthritis is worsened in IFNγ receptor-deficient mice (Vermeire et al., 1997). In addition, IFNγ knockout mice upregulate IL-1β and accelerate collagen-induced arthritis in a mouse strain resistant to developing arthritis when sensitized with collagen (Guedez et al., 2001). Some of the mechanisms by which IFNγ exerts its protective effects in these settings have been revealed, including the generation of immuno-regulatory indoleamine 2,3-dioxygenase and the conversion of $CD4^+CD25^-$ T cells to T-reg cells. Such a paradoxical role is also apparent for type 1 IFNs. During ongoing lymphocytic choriomeningitis virus infection, for instance, levels of IFNα/β persist throughout the infectious response. It emerges that, whereas early, acute production of type 1 IFNs promotes virus clearance, chronic exposure to these IFNs triggers immunosuppression via IL-10, programmed cell death ligand 1, and indolamine signaling and causes T cell apoptosis, collectively impairing the host's ability to develop specific immunity (Boasso et al., 2008; González-Navajas et al., 2012).

Thus, whereas IFNγ undoubtedly drives acute inflammation, it also dampens multiple aspects of the adaptive immune system. From our data, it appears that the signals inherent to "resolution of acute inflammation leading to adapted homeostasis" trigger the infiltration of no fewer than three cell types, including CD4, CD8, and NK cells to ensure the release of IFNγ, which, in turn triggers PGE_2 synthesis. The latter then carries out two roles—(1) it impairs further IFNγ synthesis and (2) maintains post-inflammation tolerance. We believe that the negative feedback effects of PGE_2 on IFNγ may be key in beginning to understand the complex role of this Th-1 cytokine in the dynamic continuum that is the immune response—a transient increase lasting for no more than a week (days 6–14 post-0.1 mg zymosan), during which we assume it exerts multiple (unknown) effects on various aspects of post-resolution biology, culminating in COX1/mPGEs-1 expression. Whether PGE_2 and other

Figure 6. Post-resolution PGE_2 Inhibits Adaptive Immunity

(A and B) $CD4^+$ (A) and $CD19^+$ (B) cells were FACS sorted from the post-resolving cavity (day 14) and incubated with increasing concentrations of PGE_2 equivalent to that found in the peritoneum at the same time (see Figure 2A), with/without EP receptor antagonists.

(C and D) The (C) impact of dosing mice with an EP4 receptor antagonist (MF-498 from day 6 until day 21) on T cell numbers in situ as well as their (D) phenotype as determined by intracellular IFNγ.

(E and F) Inhibiting PGE_2 synthesis (E) reduces numbers of myeloid-derived suppressor cells (sub-panel C) while increasing numbers of (F) dendritic cells in the peritoneum at day 14.

(G) Taking this further, methylated BSA (mBSA) was injected into the peritoneum or naive mice as well as those bearing a 0.1 mg zymosan at day 14; controls for this experiment were wild-type mice sensitized with complete Freund's adjuvant containing mBSA with recall assays carried out on T/B cells.

*$p \leq 0.05$; **$p \leq 0.01$; ***$p \leq 0.001$. Data are expressed as mean ± SEM; n = 6 mice/group.

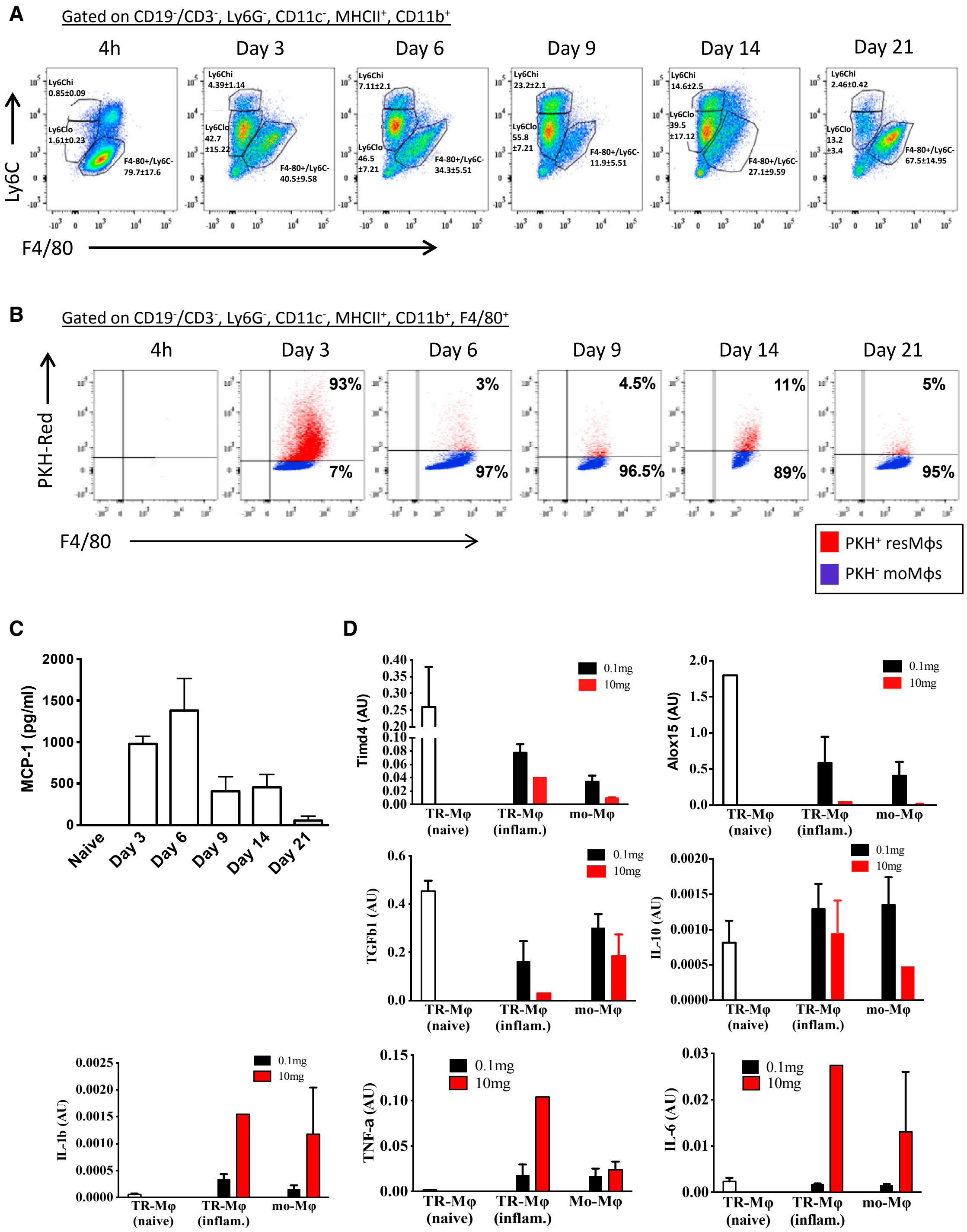

(Figure 7 continued on next page)

prostanoids are the eventual effector molecules of IFNγ's protective role in collagen-induced arthritis and experimental autoimmune encephalomyelitis remains to be investigated.

PGE_2 is erroneously thought of as purely pro-inflammatory, largely, we suspect, due to its association with nonsteroidal anti-inflammatory drugs (NSAIDs) (Moncada and Vane, 1978). Whereas the latter are certainly anti-inflammatory (Abramson et al., 1985) and undoubtedly do inhibit COX enzyme activity (Ferreira et al., 1971; Flower et al., 1972; Vane, 1971), NSAIDs possess myriad other anti-inflammatory properties aside from

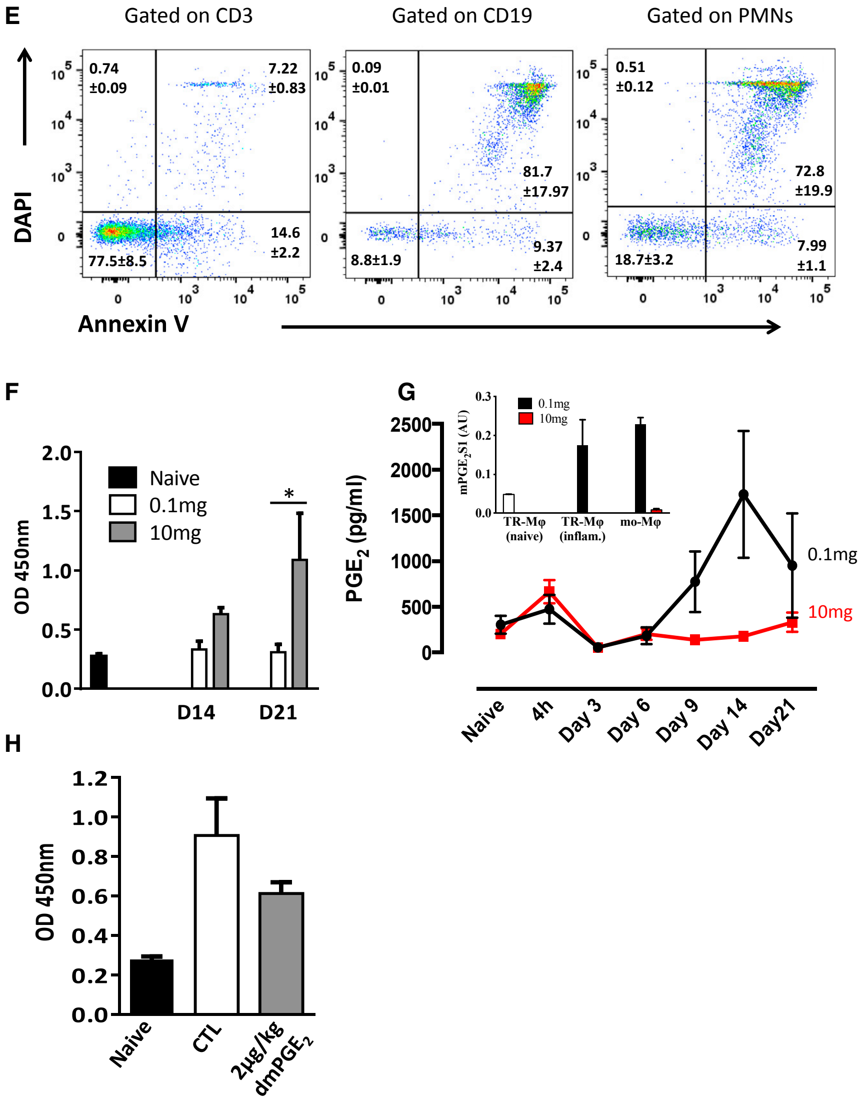

Figure 7. Autoantibodies Generated by Inflammation Driven by 10 mg Zymosan Are Inhibited by PGE_2

(A) Wild-type mice were injected with 10 mg zymosan and subjected to polychromatic flow cytometry at the indicated time points for the determination of cells of the monocyte/macrophage lineage.

(B) Following the injection of cell-tracker dyes (PKH red) into the naive peritoneum, we were able to discern tissue-resident from infiltrating monocyte-derived macrophages (mo-Mϕ).

(C and D) Levels of the (C) classic monocyte chemoattractant MCP-1 were determined in the peritoneal fluid, whereas at day 14 post-zymosan, (D) tissue-resident from infiltrating monocyte-derived macrophages were FACS sorted for the determination of their phenotype by PCR.

(E–G) At this time point, profiles of (E) apoptotic lymphocytes and granulocytes are shown alongside (F) serum levels of antibodies to dsDNA occurring in the absence of (G) peritoneal PGE_2, effects that were reversed when (H) mice receiving 10 mg zymosan were dosed from days 6 to 21 with a stable PGE_2 analog. Data are expressed as mean ± SEM; n = 6 mice/group.

COX inhibition (Abramson and Weissmann, 1989). With this in mind, we wish to put the role of PGE_2 in immunity into perspective. Unarguably, PGE_2 does cause pain and edema. However, it also suppresses bacterial phagocytosis (Aronoff et al., 2004, 2009; Medeiros et al., 2009) and NADPH-mediated bacterial killing (Serezani et al., 2005, 2007) as well as directly inhibiting T cell proliferation (Baker et al., 1981; Betz and Fox, 1991) while driving myeloid-derived suppressor cell formation (Mao et al., 2014; Obermajer and Kalinski, 2012; Sinha et al., 2007), in which case PGE_2 exerts multiple modulatory effects on innate and adaptive immunity with its predominant effect/s most likely being context dependent. With respect to our current findings, we report that, whereas PGE_2 opens up a window of local infectious opportunity, this is done in order to minimize the development of autoimmune disease, a lesser of two evils, as it were.

The conventional wisdom is that COX-2 is required for robust and substantially elevated prostanoid synthesis, such as that made during inflammation, whereas COX-1 makes prostanoids

at comparatively lower levels for the purpose of maintaining normal gut and renal physiology (Simmons et al., 2004). However, we found particularly high levels of prostanoids at day 14 derived from COX-1 with COX-2 being absent; in fact, these levels were higher than we have ever noted in animal model of inflammation. It transpires that these post-resolution prostanoids are most likely synthesized by IFNγ-induced mPGES-1, though a contribution from mPGES-2 cannot be ruled out. We do not see this phase of prolonged PGE_2 and indeed prostacyclin synthesis as being pathogenic, as animals in the post-resolution phase do not exhibit signs of discomfort or pain, events driven by PGE_2 (Kawabata, 2011) and PGI_2 (Schuh et al., 2014). Indeed, it would be of great interest to understand the endogenous mechanisms that counter-regulate the effects of these nociceptive lipid mediators in the peritoneum at day 14 post-zymosan and speculate how these protective pathways might become dysregulated during chronic pain.

In summary, we report on a sequence of events specific to resolution of acute inflammation, leading to "adapted homeostasis" that are essential for the maintenance of immune tolerance to endogenous antigens. We propose that this COX-1/mPGES axis is an internal checkpoint central to preventing the development of diseases driven by autoimmunity, which may be dysregulated in individuals with a propensity to developing chronic inflammation or subverted by infectious stimuli known to cause chronic inflammation.

EXPERIMENTAL PROCEDURES

Flow Cytometry

Flow cytometry and cell sorting were done on the LSR-Fortessa and FACSAria (BD Biosciences), respectively. Cells were incubated in FACS buffer (5% heat inactivated fetal bovine serum [FBS] [Life Technologies], 2 mM EDTA [Sigma]) in PBS (Life Technologies) with fluorescent-labeled antibodies. Data were analyzed with Flow-Jo 10.2 software (Tree Star) using fluorescent minus one controls for setting gates. Antibodies for mouse studies were obtained from BD Biosciences (Ly6C, CD11b, NK1.1, and CD8), eBioscience (Ki67, F4/80, Foxp3, and CD115), and BioLegend (Ly6G, CD3, CD19, CD4, CD44, CD62L, CD11c, major histocompatibility complex [MHC]-II, IFN-g, tumor necrosis factor [TNF], IL-10, IL-6, immunoglobulin G1 [IgG1], and IgG2a). For intracellular staining, 600,000 cells were incubated in DMEM containing penicillin/streptomycin, 10% FBS, and 2 mM L-glutamine (all Life Technologies) with 10 μg/mL brefeldin A (Sigma), 5 ng/mL phorbol 12-myristate 13-acetate (PMA) (Sigma), and 500 ng/mL ionomycin (Cayman Chemical) or 1 μg/mL lipopolysaccharide (LPS) (Sigma) for 4 hr. Cells were stained with extra cellular markers and then washed and incubated with Fix/Perm (eBioscience) and washed and incubated with Permwash (eBioscience) and fluorescent-labeled antibodies (IFNγ).

Cytokine Measurements

Cell-free exudates were measured for cytokines with a R&D Systems Luminex screening assay according to the manufacturer's instructions.

PCR

Sorted cell populations were subjected to RNA extraction using the RNeasy micro-kit (QIAGEN) according to the manufacturer's instructions. Contaminating DNA was removed by DNase I (QIAGEN) treatment. Real-time PCR was performed after 500 ng of RNA was reverse transcribed. A total of 3 ng cDNA was analyzed by quantitative real-time PCR (Applied Biosystems 7900HT) and quantified by power SYBR Green (Applied Biosystems) according to the manufacturer's instructions. For data analysis, the comparative threshold cycle values for constitutively expressed cyclophilin were used to normalize loading variations and are expressed as a.u.

Animals, Drugs, and Cell-Tracking Studies

Male C57Bl6/J mice (aged 8–10 weeks) were maintained in accordance with UK Home Office regulations (project license number P69E3D849; establishment license number X7069SDD). Peritonitis was induced by injecting sonicated 0.1 or 10 mg/mouse zymosan A (Sigma) intraperitoneally; 40,000 colony-forming units (CFU) *Streptococcus pneumoniae*$^{ova323-339}$/mouse were injected at the times indicated. *Streptococcus pneumoniae*$^{ova323-339}$ was obtained from Gerry Brown, University College London (UCL). PKH26-PCL^{red} (350 μL of 0.5 μM; Sigma) was injected intraperitoneally 2 hr prior to induction of peritonitis. Naproxen was either given (10 mg/kg; Sigma) orally in gum tragacanth twice a day from day 6 to day 14 or 21 or 20 mg/kg in drinking water from day 6 to day 21 or day 28. MF498 in gum tragacanth (30 mg/kg; Cayman Chemical) was given orally from day 6 to day 14. Bacterial handling, growth, and animal inoculation were carried out as previously described (Stables et al., 2010).

Animal Sensitization Studies

Mice were injected with 0.1 mg zymosan intraperitoneally and dosed with 20 mg/kg naproxen in drinking water from day 6 to day 21 or 28. On day 14, mice were injected intraperitoneally with 10 mg/mL methylated BSA (mBSA) (Sigma). Additionally, naive mice were injected with 10 mg/mL mBSA in complete Freund's adjuvant subcutaneously (Sigma) and left for 10 days. Bone-marrow-derived dendritic cells (DCs) were generated as previously described, and 60,000 per well were incubated overnight with 100 ng/mL LPS and 20 ng/mL Met BSA. DCs were washed and incubated with 300,000 lingual lymph node cells in RPMI (Life Technologies) with 30 U/mL IL-2 (Miltenyi Biotec) for 4 days. Cells were stained for FACS with CD11b, F480, CD11c, MHCII, CD19, CD3, CD4, CD8, and Ki67.

Measurements of dsDNA

Blood was taken by cardiac puncture, the blood was left to clot, and the serum stored at -80°C until analysis. High binding plates were (Costar; Appleton Woods) first coated with 20 μg/mL of poly-L-lysine (Sigma) and then 20 μg/mL calf thymus DNA (Sigma). Serum was diluted 1:100 with 1% BSA (Sigma) in PBS for 1 hr at room temperature. Plates were washed and incubated with goat anti-mouse IgG-horseradish peroxidase (HRP) (Thermo Scientific). Data are expressed as optical density.

Lipidomics

COX- and LOX-derived lipid mediators were extracted from cell-free inflammatory exudates as previously described (Massey and Nicolaou, 2013). Briefly, samples were defrosted on ice and then diluted to 4 mL at a final concentration of 15% (v/v) methanol/water. Internal standards were added (20 ng each of PGB_2-*d*4, 12-HETE-*d*8, 8,9DHET-*d*11, and 8(9)EET-*d*11; Cayman Chemical, Ann Arbor, USA). Semi-purification of samples was performed using solid-phase extraction (C18-E cartridges; Phenomenex, Macclesfield, UK), and lipid mediators were eluted using methyl formate. Analytes were separated on a C18 column (Acquity UPLC BEH; 1.7 μm; 2.1 × 50 mm; Waters, Wilmslow, UK) using ultraperformance liquid chromatography (Acquity; Waters, Wilmslow, UK) coupled to a triple quadrupole mass spectrometer with electrospray ionization (Xevo TQ-S; Waters, Wilmslow, UK). Analytes were quantified using multiple reaction monitoring and calibration lines constructed using commercially available standards (Cayman Chemicals, Ann Arbor, USA).

Cell Culture

Naive peritoneal washouts were spun down at 500 g for 5 min to separate cells from inflammatory exudate. Cells were then resuspended in ACK lysis buffer (Lonza) to remove red blood cells for 30 s, after which they were diluted with FACS buffer and spun as above. Cells were resuspended in MACS buffer and counted and incubated with anti-mouse CD19 beads (Miltenyi Biotec) for 15 min. The labeled cells were washed and passed through an MS column (Miltenyi Biotec). The flowthrough was plated out at 450,000 cells per well on a 24-well plate. Cells were left to adhere for 30 min, after which non-adherent cells were washed off. Naive macrophages were incubated with 100 ng/mL TNFα, 250 pg/mL IFNγ, 300 pg/mL IP-10, and 300 pg/mL MIG (Peprotech)

for 24 hr. Cell supernatants were stored at −80°C, and cells were stored at −80°C in RIPA buffer for analysis by western blot.

Western Blotting

Western blotting was carried out as previously described (Newson et al., 2014). Briefly, cells from peritoneal washouts or ex vivo culture were lysed in RIPA buffer with protease inhibitors (both Sigma) and the protein concentration determined by Bradford assay (Bio-Rad). Ten micrograms of protein were separated by SDS-PAGE (National Diagnostics). Separated proteins were transferred onto a polyvinylidene fluoride membrane (Immobilon; Millipore) and incubated with COX-1, COX-2, mPGES-1, mPGE-2, EP1-4 (Cayman Chemical), β-actin, and GAPDH (Sigma) overnight in block buffer (Tris-HCL, 1% Tween-20, 1% BSA [Sigma], and 5% nonfat milk [Marvel]). Blots were washed and incubated with HRP-conjugated antibodies (Santa Cruz Biotechnology) for 1 hr at room temperature in blocking buffer. Specific proteins were visualized by enhanced chemiluminescence (ECL) hyperfilm.

Murine Sickness Score

Mice injected with *Streptococcus pneumoniae* were monitored at the times indicated in the results. Each mouse was scored for levels of sickness based on the following scoring system. If a mouse showed signs of piloerection, slow movement, and a hunched posture, then a score of 1 was given. If a mouse displayed 2 of these parameters, then a score of 2 was given. Mice showing all three of these clinical signs or pus in eyes were given a score of 3. Failure to move was scored at 4, at which point the mice were killed.

Statistical Analysis

For comparisons between multiple groups, 1-way ANOVA with repeated measures was performed followed by Bonferroni post-test. Comparisons between 2 groups were made by 2-tailed (un)paired t test. Data are presented as mean ± SEM with numbers of animals used per experiment stipulated accordingly.

SUPPLEMENTAL INFORMATION

Supplemental Information includes four figures and can be found with this article online at http://dx.doi.org/10.1016/j.celrep.2017.08.098.

AUTHOR CONTRIBUTIONS

J.N., along with MS, carried out the majority of the animal experiments, including ex vivo assays and flow cytometry. M.A. and G.G.M. carried out PCR assays; A.N. and A.C.K. carried out lipidomic analysis, whereas M.B., M.P.M., and R.V.D.M. carried out ELISA assays. D.W.G., A.N., and R.P.H.D.M. had substantial academic input and contributed to study design. D.W.G. directed and coordinated the research and wrote the paper. All authors participated in critical revisions. S.J. made the serendipitous observation of PGE_2 being elevated post-resolution; this was done during a summer project in the lab of D.W.G.

ACKNOWLEDGMENTS

This work was funded by the Wellcome Trust by a Senior Research Fellowship (WT087520) awarded to D.W.G.

Received: May 2, 2017
Revised: July 18, 2017
Accepted: August 28, 2017
Published: September 26, 2017

REFERENCES

Abramson, S.B., and Weissmann, G. (1989). The mechanisms of action of nonsteroidal antiinflammatory drugs. Arthritis Rheum. *32*, 1–9.

Abramson, S., Korchak, H., Ludewig, R., Edelson, H., Haines, K., Levin, R.I., Herman, R., Rider, L., Kimmel, S., and Weissmann, G. (1985). Modes of action of aspirin-like drugs. Proc. Natl. Acad. Sci. USA *82*, 7227–7231.

Aronoff, D.M., Canetti, C., and Peters-Golden, M. (2004). Prostaglandin E2 inhibits alveolar macrophage phagocytosis through an E-prostanoid 2 receptor-mediated increase in intracellular cyclic AMP. J. Immunol. *173*, 559–565.

Aronoff, D.M., Lewis, C., Serezani, C.H., Eaton, K.A., Goel, D., Phipps, J.C., Peters-Golden, M., and Mancuso, P. (2009). E-prostanoid 3 receptor deletion improves pulmonary host defense and protects mice from death in severe Streptococcus pneumoniae infection. J. Immunol. *183*, 2642–2649.

Baker, P.E., Fahey, J.V., and Munck, A. (1981). Prostaglandin inhibition of T-cell proliferation is mediated at two levels. Cell. Immunol. *61*, 52–61.

Betz, M., and Fox, B.S. (1991). Prostaglandin E2 inhibits production of Th1 lymphokines but not of Th2 lymphokines. J. Immunol. *146*, 108–113.

Boasso, A., Hardy, A.W., Anderson, S.A., Dolan, M.J., and Shearer, G.M. (2008). HIV-induced type I interferon and tryptophan catabolism drive T cell dysfunction despite phenotypic activation. PLoS ONE *3*, e2961.

Dinauer, M.C. (1993). The respiratory burst oxidase and the molecular genetics of chronic granulomatous disease. Crit. Rev. Clin. Lab. Sci. *30*, 329–369.

Ferber, I.A., Brocke, S., Taylor-Edwards, C., Ridgway, W., Dinisco, C., Steinman, L., Dalton, D., and Fathman, C.G. (1996). Mice with a disrupted IFN-gamma gene are susceptible to the induction of experimental autoimmune encephalomyelitis (EAE). J. Immunol. *156*, 5–7.

Ferreira, S.H., Moncada, S., and Vane, J.R. (1971). Indomethacin and aspirin abolish prostaglandin release from the spleen. Nat. New Biol. *231*, 237–239.

Flower, R., Gryglewski, R., Herbaczyńska-Cedro, K., and Vane, J.R. (1972). Effects of anti-inflammatory drugs on prostaglandin biosynthesis. Nat. New Biol. *238*, 104–106.

Fonseca, D.M., Hand, T.W., Han, S.J., Gerner, M.Y., Glatman Zaretsky, A., Byrd, A.L., Harrison, O.J., Ortiz, A.M., Quinones, M., Trinchieri, G., et al. (2015). Microbiota-dependent sequelae of acute infection compromise tissue-specific immunity. Cell *163*, 354–366.

Fujinami, R.S., Oldstone, M.B., Wroblewska, Z., Frankel, M.E., and Koprowski, H. (1983). Molecular mimicry in virus infection: crossreaction of measles virus phosphoprotein or of herpes simplex virus protein with human intermediate filaments. Proc. Natl. Acad. Sci. USA *80*, 2346–2350.

Fujinami, R.S., von Herrath, M.G., Christen, U., and Whitton, J.L. (2006). Molecular mimicry, bystander activation, or viral persistence: infections and autoimmune disease. Clin. Microbiol. Rev. *19*, 80–94.

González-Navajas, J.M., Lee, J., David, M., and Raz, E. (2012). Immunomodulatory functions of type I interferons. Nat. Rev. Immunol. *12*, 125–135.

Guedez, Y.B., Whittington, K.B., Clayton, J.L., Joosten, L.A., van de Loo, F.A., van den Berg, W.B., and Rosloniec, E.F. (2001). Genetic ablation of interferon-gamma up-regulates interleukin-1beta expression and enables the elicitation of collagen-induced arthritis in a nonsusceptible mouse strain. Arthritis Rheum. *44*, 2413–2424.

Jamieson, T., Cook, D.N., Nibbs, R.J., Rot, A., Nixon, C., McLean, P., Alcami, A., Lira, S.A., Wiekowski, M., and Graham, G.J. (2005). The chemokine receptor D6 limits the inflammatory response in vivo. Nat. Immunol. *6*, 403–411.

Kalinski, P. (2012). Regulation of immune responses by prostaglandin E2. J. Immunol. *188*, 21–28.

Kawabata, A. (2011). Prostaglandin E2 and pain–an update. Biol. Pharm. Bull. *34*, 1170–1173.

Kuperman, D.A., Huang, X., Koth, L.L., Chang, G.H., Dolganov, G.M., Zhu, Z., Elias, J.A., Sheppard, D., and Erle, D.J. (2002). Direct effects of interleukin-13 on epithelial cells cause airway hyperreactivity and mucus overproduction in asthma. Nat. Med. *8*, 885–889.

Lawrence, T., Willoughby, D.A., and Gilroy, D.W. (2002). Anti-inflammatory lipid mediators and insights into the resolution of inflammation. Nat. Rev. Immunol. *2*, 787–795.

León, B., López-Bravo, M., and Ardavín, C. (2007). Monocyte-derived dendritic cells formed at the infection site control the induction of protective T helper 1 responses against Leishmania. Immunity *26*, 519–531.

Lykens, J.E., Terrell, C.E., Zoller, E.E., Divanovic, S., Trompette, A., Karp, C.L., Aliberti, J., Flick, M.J., and Jordan, M.B. (2010). Mice with a selective

impairment of IFN-gamma signaling in macrophage lineage cells demonstrate the critical role of IFN-gamma-activated macrophages for the control of protozoan parasitic infections in vivo. J. Immunol. *184*, 877–885.

Mao, Y., Sarhan, D., Steven, A., Seliger, B., Kiessling, R., and Lundqvist, A. (2014). Inhibition of tumor-derived prostaglandin-e2 blocks the induction of myeloid-derived suppressor cells and recovers natural killer cell activity. Clin. Cancer Res. *20*, 4096–4106.

Massey, K.A., and Nicolaou, A. (2013). Lipidomics of oxidized polyunsaturated fatty acids. Free Radic. Biol. Med. *59*, 45–55.

Medeiros, A.I., Serezani, C.H., Lee, S.P., and Peters-Golden, M. (2009). Efferocytosis impairs pulmonary macrophage and lung antibacterial function via PGE2/EP2 signaling. J. Exp. Med. *206*, 61–68.

Moncada, S., and Vane, J.R. (1978). Pharmacology and endogenous roles of prostaglandin endoperoxides, thromboxane A2, and prostacyclin. Pharmacol. Rev. *30*, 293–331.

Morgenstern, D.E., Gifford, M.A., Li, L.L., Doerschuk, C.M., and Dinauer, M.C. (1997). Absence of respiratory burst in X-linked chronic granulomatous disease mice leads to abnormalities in both host defense and inflammatory response to Aspergillus fumigatus. J. Exp. Med. *185*, 207–218.

Nakano, H., Lin, K.L., Yanagita, M., Charbonneau, C., Cook, D.N., Kakiuchi, T., and Gunn, M.D. (2009). Blood-derived inflammatory dendritic cells in lymph nodes stimulate acute T helper type 1 immune responses. Nat. Immunol. *10*, 394–402.

Nathan, C. (2002). Points of control in inflammation. Nature *420*, 846–852.

Newson, J., Stables, M., Karra, E., Arce-Vargas, F., Quezada, S., Motwani, M., Mack, M., Yona, S., Audzevich, T., and Gilroy, D.W. (2014). Resolution of acute inflammation bridges the gap between innate and adaptive immunity. Blood *124*, 1748–1764.

O'Brien, A.J., Fullerton, J.N., Massey, K.A., Auld, G., Sewell, G., James, S., Newson, J., Karra, E., Winstanley, A., Alazawi, W., et al. (2014). Immunosuppression in acutely decompensated cirrhosis is mediated by prostaglandin E2. Nat. Med. *20*, 518–523.

Obermajer, N., and Kalinski, P. (2012). Generation of myeloid-derived suppressor cells using prostaglandin E2. Transplant. Res. *1*, 15.

Obermajer, N., Muthuswamy, R., Lesnock, J., Edwards, R.P., and Kalinski, P. (2011). Positive feedback between PGE2 and COX2 redirects the differentiation of human dendritic cells toward stable myeloid-derived suppressor cells. Blood *118*, 5498–5505.

Rajakariar, R., Hilliard, M., Lawrence, T., Trivedi, S., Colville-Nash, P., Bellingan, G., Fitzgerald, D., Yaqoob, M.M., and Gilroy, D.W. (2007). Hematopoietic prostaglandin D2 synthase controls the onset and resolution of acute inflammation through PGD2 and 15-deoxyDelta12 14 PGJ2. Proc. Natl. Acad. Sci. USA *104*, 20979–20984.

Savill, J.S., Wyllie, A.H., Henson, J.E., Walport, M.J., Henson, P.M., and Haslett, C. (1989). Macrophage phagocytosis of aging neutrophils in inflammation. Programmed cell death in the neutrophil leads to its recognition by macrophages. J. Clin. Invest. *83*, 865–875.

Schuh, C.D., Pierre, S., Weigert, A., Weichand, B., Altenrath, K., Schreiber, Y., Ferreiros, N., Zhang, D.D., Suo, J., Treutlein, E.M., et al. (2014). Prostacyclin mediates neuropathic pain through interleukin 1β-expressing resident macrophages. Pain *155*, 545–555.

Segal, A.W. (1996). The NADPH oxidase and chronic granulomatous disease. Mol. Med. Today *2*, 129–135.

Segal, A.W., and Peters, T.J. (1976). Characterisation of the enzyme defect in chronic granulomatous disease. Lancet *1*, 1363–1365.

Serezani, C.H., Aronoff, D.M., Jancar, S., Mancuso, P., and Peters-Golden, M. (2005). Leukotrienes enhance the bactericidal activity of alveolar macrophages against Klebsiella pneumoniae through the activation of NADPH oxidase. Blood *106*, 1067–1075.

Serezani, C.H., Chung, J., Ballinger, M.N., Moore, B.B., Aronoff, D.M., and Peters-Golden, M. (2007). Prostaglandin E2 suppresses bacterial killing in alveolar macrophages by inhibiting NADPH oxidase. Am. J. Respir. Cell Mol. Biol. *37*, 562–570.

Serhan, C.N., Brain, S.D., Buckley, C.D., Gilroy, D.W., Haslett, C., O'Neill, L.A., Perretti, M., Rossi, A.G., and Wallace, J.L. (2007). Resolution of inflammation: state of the art, definitions and terms. FASEB J. *21*, 325–332.

Simmons, D.L., Botting, R.M., and Hla, T. (2004). Cyclooxygenase isozymes: the biology of prostaglandin synthesis and inhibition. Pharmacol. Rev. *56*, 387–437.

Sinha, P., Clements, V.K., Fulton, A.M., and Ostrand-Rosenberg, S. (2007). Prostaglandin E2 promotes tumor progression by inducing myeloid-derived suppressor cells. Cancer Res. *67*, 4507–4513.

Stables, M.J., Newson, J., Ayoub, S.S., Brown, J., Hyams, C.J., and Gilroy, D.W. (2010). Priming innate immune responses to infection by cyclooxygenase inhibition kills antibiotic-susceptible and -resistant bacteria. Blood *116*, 2950–2959.

Stoecklin, G., and Anderson, P. (2006). Posttranscriptional mechanisms regulating the inflammatory response. Adv. Immunol. *89*, 1–37.

Taub, D.D., Lloyd, A.R., Conlon, K., Wang, J.M., Ortaldo, J.R., Harada, A., Matsushima, K., Kelvin, D.J., and Oppenheim, J.J. (1993). Recombinant human interferon-inducible protein 10 is a chemoattractant for human monocytes and T lymphocytes and promotes T cell adhesion to endothelial cells. J. Exp. Med. *177*, 1809–1814.

Teijaro, J.R., Ng, C., Lee, A.M., Sullivan, B.M., Sheehan, K.C., Welch, M., Schreiber, R.D., de la Torre, J.C., and Oldstone, M.B. (2013). Persistent LCMV infection is controlled by blockade of type I interferon signaling. Science *340*, 207–211.

Uderhardt, S., Herrmann, M., Oskolkova, O.V., Aschermann, S., Bicker, W., Ipseiz, N., Sarter, K., Frey, B., Rothe, T., Voll, R., et al. (2012). 12/15-lipoxygenase orchestrates the clearance of apoptotic cells and maintains immunologic tolerance. Immunity *36*, 834–846.

Vane, J.R. (1971). Inhibition of prostaglandin synthesis as a mechanism of action for aspirin-like drugs. Nat. New Biol. *231*, 232–235.

Vermeire, K., Heremans, H., Vandeputte, M., Huang, S., Billiau, A., and Matthys, P. (1997). Accelerated collagen-induced arthritis in IFN-gamma receptor-deficient mice. J. Immunol. *158*, 5507–5513.

Wakim, L.M., and Bevan, M.J. (2011). Cross-dressed dendritic cells drive memory CD8+ T-cell activation after viral infection. Nature *471*, 629–632.

Wilson, E.B., Yamada, D.H., Elsaesser, H., Herskovitz, J., Deng, J., Cheng, G., Aronow, B.J., Karp, C.L., and Brooks, D.G. (2013). Blockade of chronic type I interferon signaling to control persistent LCMV infection. Science *340*, 202–207.

Woodland, D.L., and Blackman, M.A. (1992). Retroviral super-antigens and T cells. Int. Rev. Immunol. *8*, 311–325.

Wright, K.L., Weaver, S.A., Patel, K., Coopman, K., Feeney, M., Kolios, G., Robertson, D.A., and Ward, S.G. (2004). Differential regulation of prostaglandin E biosynthesis by interferon-gamma in colonic epithelial cells. Br. J. Pharmacol. *141*, 1091–1097.

Yona, S., Kim, K.W., Wolf, Y., Mildner, A., Varol, D., Breker, M., Strauss-Ayali, D., Viukov, S., Guilliams, M., Misharin, A., et al. (2013). Fate mapping reveals origins and dynamics of monocytes and tissue macrophages under homeostasis. Immunity *38*, 79–91.

Cell Reports, Volume *20*

Supplemental Information

Inflammatory Resolution Triggers a Prolonged Phase of Immune Suppression through COX-1/mPGES-1-Derived Prostaglandin E_2

Justine Newson, Madhur P. Motwani, Alexandra C. Kendall, Anna Nicolaou, Giulio G. Muccioli, Mireille Alhouayek, Melanie Bennett, Rachel Van De Merwe, Sarah James, Roel P.H. De Maeyer, and Derek W. Gilroy

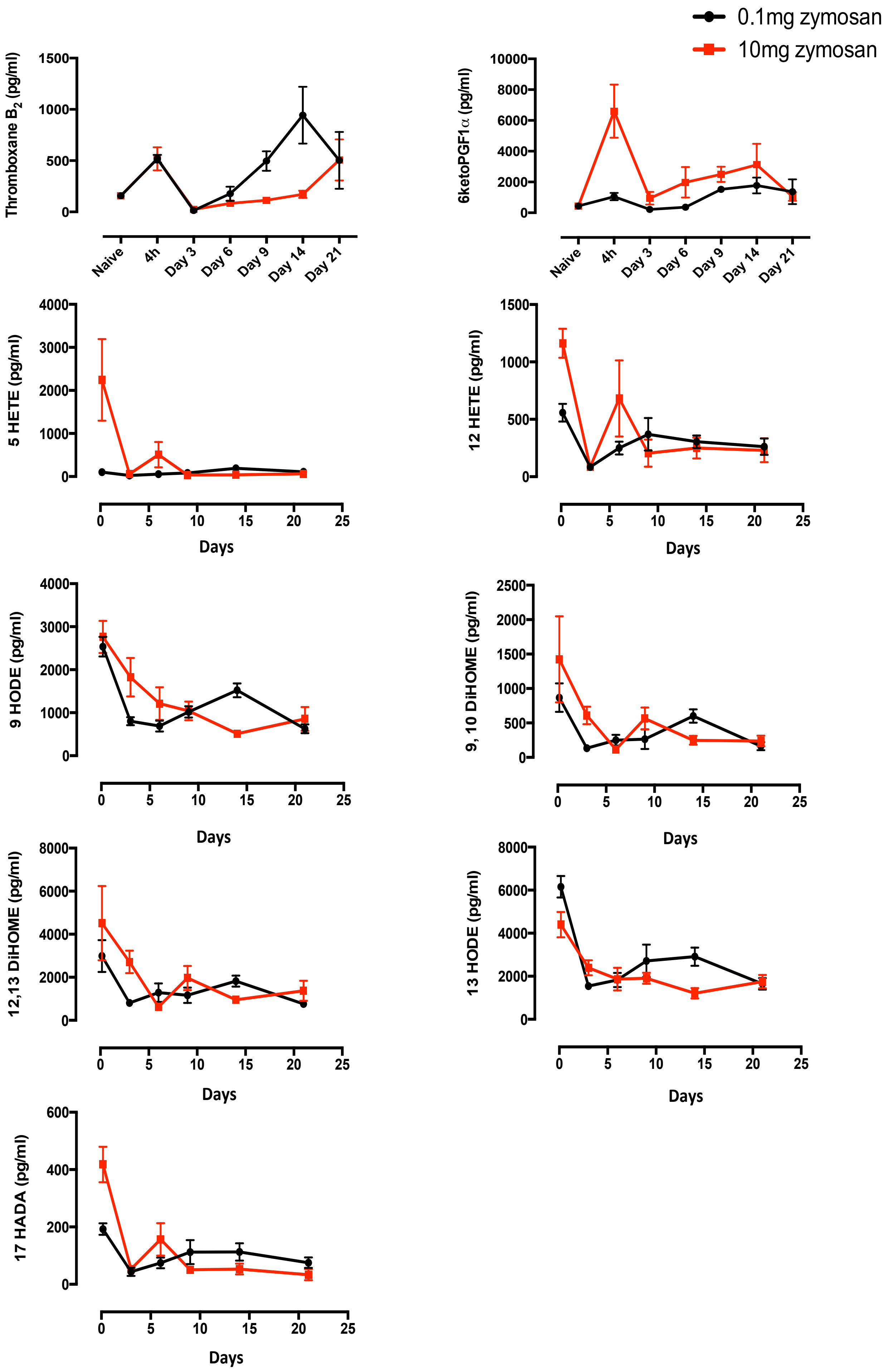

Figure S1. Lipidomic profiling of cell-free inflammatory exudate from mice that received either 0.1 or 10mg zymosan i.p. Related to Figure 2

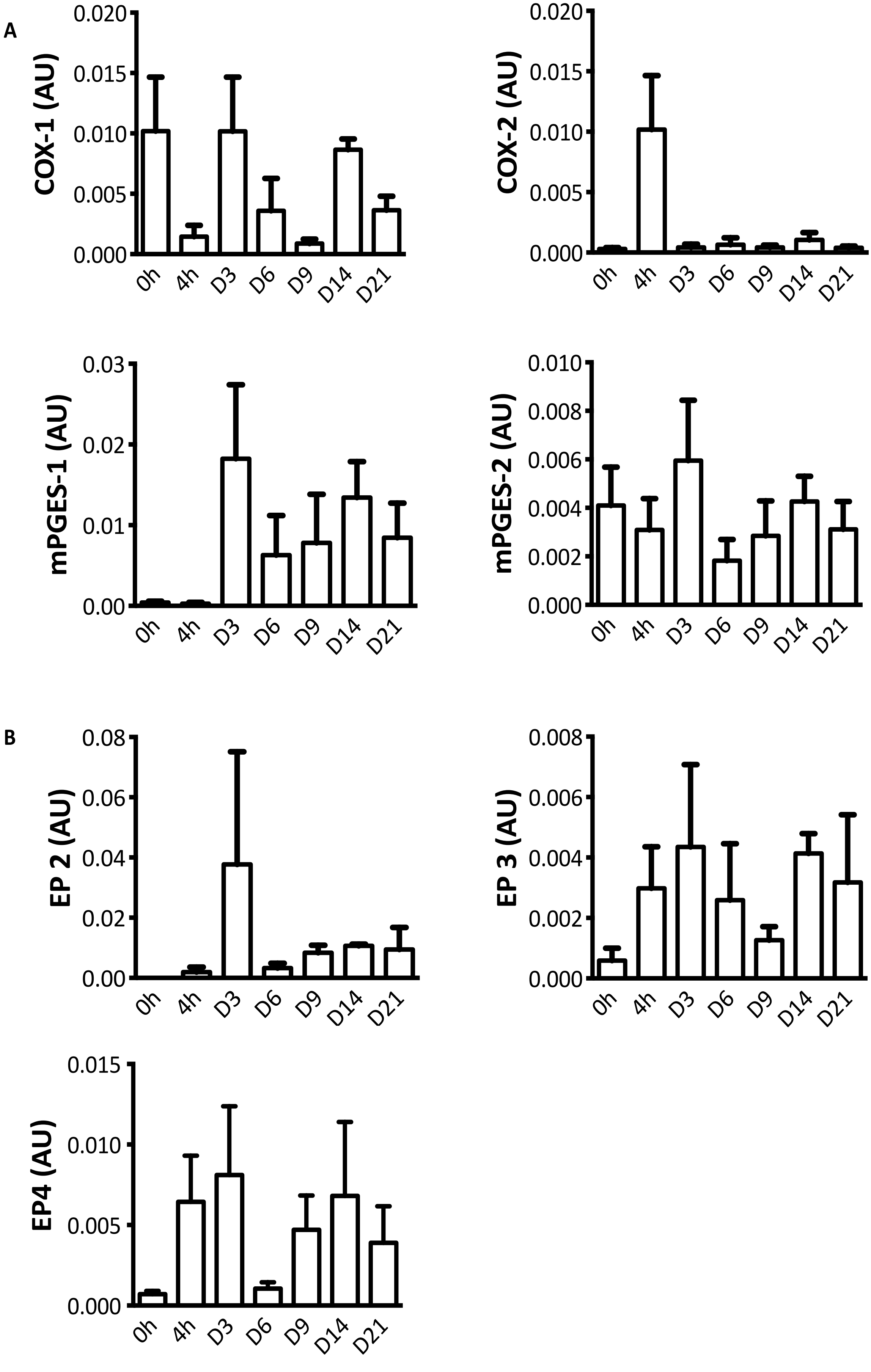

Figure S2. (A) Densitometry values for the temporal expression of COX and mPGES isoforms and (B) PGE_2 receptors on bulk cells from a mouse peritonitis driven by 0.1mg zymosan i.p. Related to Figures 2 and 3.

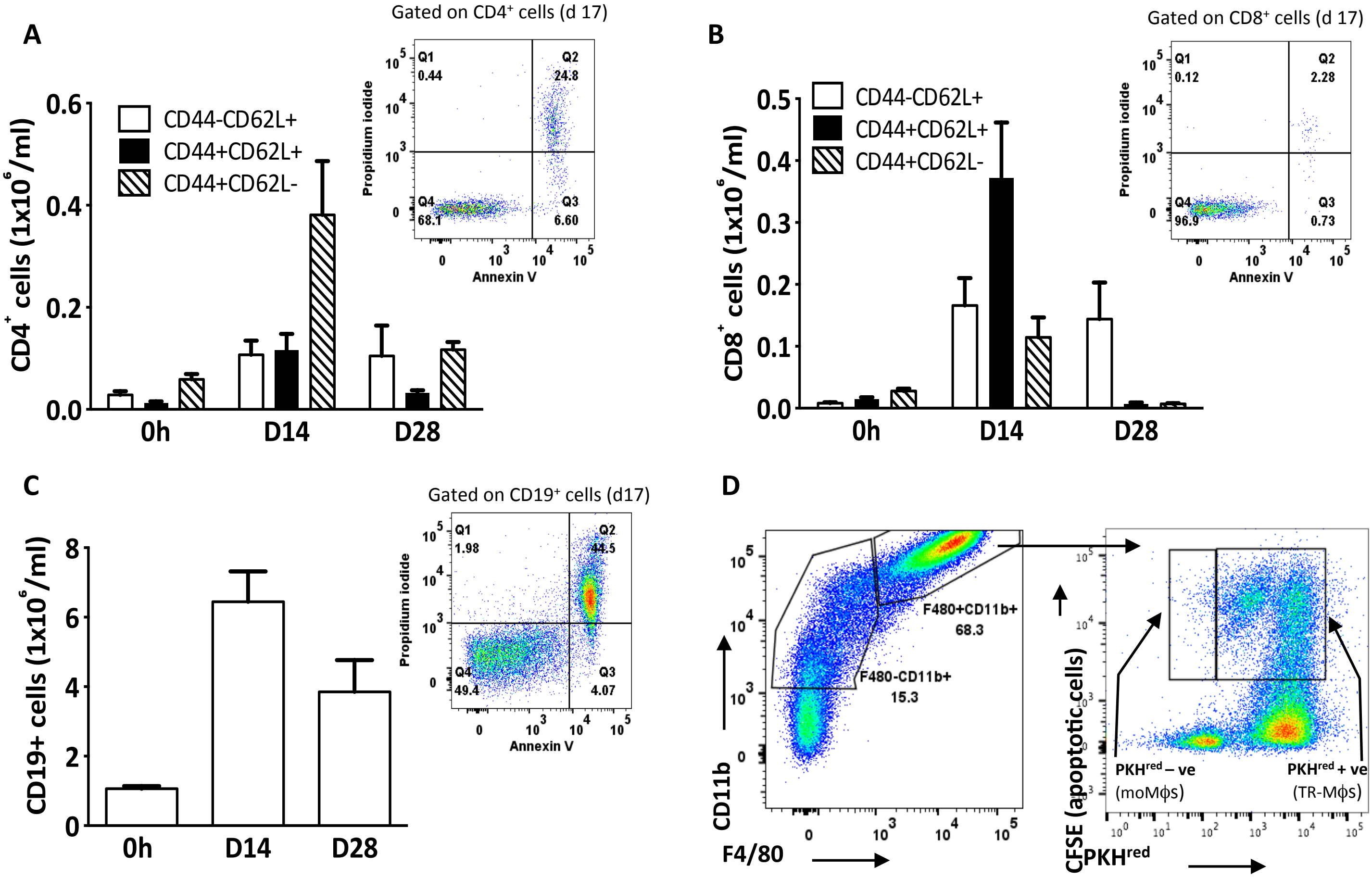

Figure S3. Expansion and contraction of **(A-B)** memory T cell populations as well as **(C)** B cells from day 14 post 0.1mg zymosan injection. The reduction in lymphocyte numbers was due, at least in part, to programmed cell death with apoptotic bodies **(insert panels to A-C)** being cleared by **(D)** tissue-resident macrophages. This assertion is derived from an experiment where we injected apoptotic lymphocytes labelled with PKH^{red} into the peritoneum at day 14 finding that PKH^{red} positive tissue-resident macrophages preferentially phagocytosed these cells and not infiltrating monocyte-derived macrophages. Related to Figures 6 and 7

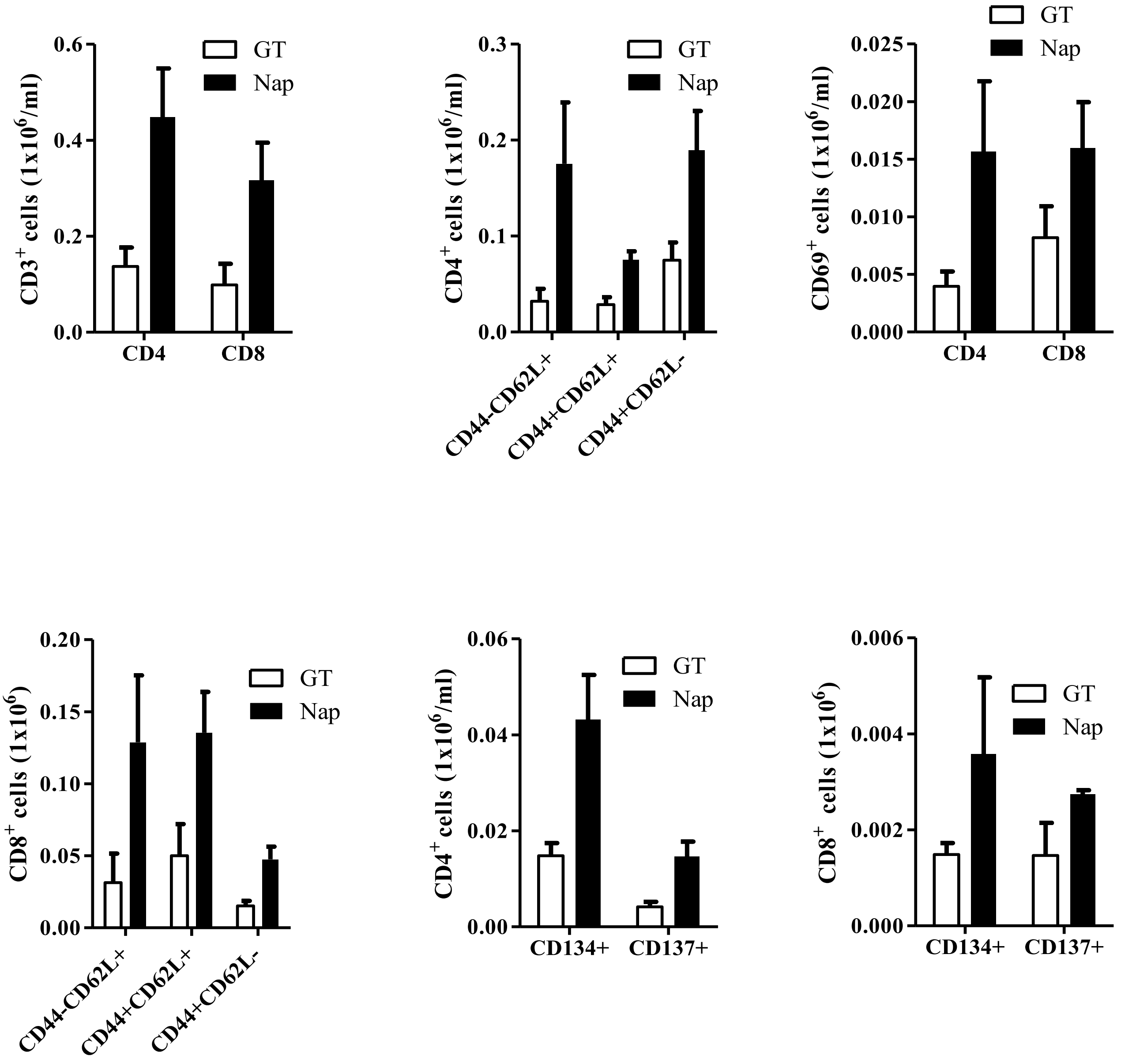

Figure S4. Effect of the non-selective COX 2 inhibitor naproxen on T cell numbers and phenotype when dosed orally to mice bearing a 0.1mg zymosan-induced peritonitis from day for two weeks. Related to Figure 6.
